# Brain tumor classification using GAN-augmented data with autoencoders and Swin Transformers

**DOI:** 10.3389/fmed.2025.1635796

**Published:** 2025-08-22

**Authors:** Abdullah Almuhaimeed, Anas Bilal, Abdulkareem Alzahrani, Malek Alrashidi, Mansoor Alghamdi, Raheem Sarwar

**Affiliations:** ^1^Digital Health Institute, King Abdulaziz City for Science and Technology, Riyadh, Saudi Arabia; ^2^College of Information Science and Technology, Hainan Normal University, Haikou, China; ^3^Department of Computer Science, Faculty of Computing and Information, Al-Baha University, Al-Baha, Saudi Arabia; ^4^Department of Computer Science, Applied College, University of Tabuk, Tabuk, Saudi Arabia; ^5^OTEHM, Manchester Metropolitan University, Manchester, United Kingdom

**Keywords:** brain tumour classification, conditional GAN, synthetic data, Swin Transformer, autoencoders

## Abstract

**Introduction:**

Brain tumor classification remains one of the most challenging tasks in medical image analysis, with diagnostic errors potentially leading to severe consequences. Existing methods often fail to fully exploit all relevant features, focusing on a limited set of deep features that may miss the complexity of the task.

**Methods:**

In this paper, we propose a novel deep learning model combining a Swin Transformer and AE-cGAN augmentation to overcome challenges such as data imbalance and feature extraction. AE-cGAN generates synthetic images, enhancing dataset diversity and improving the model’s generalization. The Swin Transformer excels at capturing both local and global dependencies, while AE-cGAN generates synthetic data that enables classification of multiple brain tumor morphologies.

**Results:**

The model achieved impressive accuracy rates of 99.54% and 98.9% on two publicly available datasets, Figshare and Kaggle, outperforming state-of-the-art methods. Our results demonstrate significant improvements in classification, sensitivity, and specificity.

**Discussion:**

These findings indicate that the proposed approach effectively addresses data imbalance and feature extraction limitations, leading to superior performance in brain tumor classification. Future work will focus on real-time clinical deployment and expanding the model’s application to various medical imaging tasks.

## 1 Introduction

Recent progress in medical imaging and computational intelligence has demonstrated the power of deep learning, multimodal fusion, and quantum-inspired methods in solving complex clinical problems across diverse medical domains. For instance, image-guided tract-based surgical approaches have improved hematoma evacuation outcomes for intracerebral hemorrhage (1). EEG-based frameworks have shown remarkable success in visual stimulus reconstruction (2), mental state estimation (3), and emotion recognition via autoencoder fusion with transformer models like MSC-TimesNet (4). Multimodal masked autoencoders and lightweight modules such as AFBNet have also emerged to address the challenges of feature extraction and computational efficiency in disease staging and super-resolution imaging (5, 6). Biologically inspired strategies, like miRNA-guided neuroregeneration, are being computationally modeled to support post-hemorrhagic remyelination (7). Transformer-driven networks such as CenterFormer are transforming segmentation accuracy in dental imaging, while CI-based systems enhance neuropsychological assessment of substance-related disorders (8, 9). Additionally, deep learning has accelerated super-resolution microvessel imaging and denoising in ultrasound microscopy, addressing long-standing imaging limitations. Advanced modeling is also being applied to rare neurological diseases, like adult-onset ceroid lipofuscinosis, and to cross-modal causal learning frameworks in radiology report generation (10–13). Furthermore, recent efforts have explored spectral graph signal processing to better model brain connectivity in autism (14) learning techniques have shown strong potential in early multi-cancer detection (15), breast cancer classification using SqueezeNet-SVM (16), efficient dual-encoder segmentation in dermatology (17), and hybrid ELM models for breast cancer diagnosis (18). These innovations reflect a growing consensus that next-generation diagnostic systems must integrate robust data augmentation, multimodal fusion, and advanced architectures like transformers to deliver clinical-grade accuracy and generalizability.

One area where these advancements are critically needed is in the diagnosis and classification of brain tumours, a domain characterized by high complexity, diagnostic uncertainty, and significant clinical risk. Brain tumours are among the most intricate and life-threatening neurological conditions, and the patient’s health outcomes and quality of life are greatly impacted (19). A brain tumour is an umbrella term that describes the abnormal and often uncontrolled division of cells in the brain and central nervous system. Brain tumours can be benign, malignant or metastatic (having spread from other regions of the body). Primary brain tumours are those that originate from within the brain. Examples of primary brain tumours are gliomas, meningiomas, and pituitary adenomas (20, 21). The associated symptoms of a brain tumour will vary greatly depending on the size and location. Common associated symptoms include speech, vision, or sensation changes, coordination problems, cognitive changes, recurrent headaches, and seizures (22, 23). Diagnosing brain tumours is oftentimes difficult due to the heterogeneous way they present and the often-subtle nature of the early-stage symptoms (24, 25). These factors often result in delays in treatment, increased complexity of health outcomes, and increased mortality.

To address these issues, computer-assisted detection and diagnosis (CADe and CADx) systems have developed as essential tools within medical imaging by providing valuable support in detecting and classifying abnormalities to radiologists. Such systems rely on a combination of artificial intelligence (AI) and computer vision methods that can analyse medical images, for example, colorectal polyp segmentation, lung cancer detection, tumour classification (26–28). Machine learning (ML) methods, especially deep learning, have transformed this field by applying models that can automatically learn features, find complex patterns and improve diagnostic accuracy (29, 30). Furthermore, CADe and CADx systems offer not only a more accurate diagnosis, but also further reproducible and efficient levels of accuracy which ultimately is crucial in clinical practice where timely and accurate decisions can save lives (31). Notwithstanding advances made in ML and deep learning, a main bottleneck in brain tumour classification studies is the limited number of available high-quality annotated examples. Deep learning models, in particular, Convolutional Neural Networks (CNN), have performed very well by learning hierarchical spatial features automatically from medical images. However, it is the case that deep learning relies heavily on and requires considerable datasets that are very high in both volume and quality. On the other hand, data augmentation strategies, like image flipping, rotation, or cropping, will achieve limited degrees of diversity and have their limitations in capturing the full spectrum of pathological variations expected in real medical data.

The use of ML and DL approaches for the classification of brain tumours has received a lot of attention. Early work in this area used traditional, ML-based approaches that started with manually determining features and then using the features in traditional classifiers (32) created a feature set by combining the Gray Level Co-occurrence Matrix (GLCM) and Principal Component Analysis (PCA) for feature selection before applying classifiers including SVM, KNN along with Generalized Regression Neural Networks (GRNN). They achieved accuracy of 97, 96.24 and 94.7%, respectively. Although these studies reported good classification results, almost every step depended on handcrafted features that probably did not completely represent the full complexity of the underlying tumour structures shown in MRI images. Such features are likely to cause problems with generalization on unseen data and raise concerns related to robustness in clinical practice.

With the rise of DL, models such as CNN have created a means for automating both deciding on the features to use, and then classifying features (33) completed an evaluation of multiple transfer learning models, including ResNet, Xception, and MobileNetV2, and applied these models to brain tumour detection. Out of the previously listed models, MobileNetV2 had accuracy of 98.24% accuracy and was determined to offer a powerful balance between performance and computational efficient. MobileNetV2 was efficient, but may not be able to extract as much features to measure complex tumours as other architectures would offer, such as ResNet or Xception, and have offered superior accuracy at the cost of speed. This tradeoff between model size and computational efficient have been a large factor in realizing the model for real-time systems and especially in clinical systems (34), compared AlexNet, VGG16, GoogLeNet, and ResNet-50, on a dataset of 3,000 MRI scans. ResNet-50, had the highest accuracy of the previous category, utilizing 95.8% accuracy, where AlexNet, the fastest model and completed training in 1.2 s, no less. While AlexNet is quick, how close it came to the final weighted estimate associated with tasks that it is asked to complete, and therefore, will be inhibitive in spaces where super high classification accuracy is required, such as when medical diagnoses where critically every detail matters.

New advancements were found when attention mechanisms were embedded within CNNs to concentrate on key features of the input data (35) made modifications to GoogLeNet by adding attention layers and residual connections to the network, increasing accuracy by 1.72% while retaining fewer parameters than the originally designed GoogLeNet. This model without attention mechanisms works well for a variety of tasks, and this newly modified solution has the potential of further improving performance measures, but the additional computational burden in terms of the residual connections and attention layers could still fail to guarantee any efficiency with real-time performance in resource-constrained environments (36) adapted attention mechanisms into a VGG-based architecture (AVNC) for the classification of chest CT images, and there was demonstrated better performance, but due to the need for additional memory (and complexity), real-time deployment may not be practicable. Combining various deep learning techniques into hybrid models has had much success improving classification accuracy in recent years. For instance, (37) proposed a hybrid CNN combined with an LSTM. R. Vankdothu et al. built a hybrid CNN-LSTM by performing feature extraction with their CNN before passing the result to the LSTM to capture contextual information. The drawback to creating a hybrid CNN-LSTM is the computational overhead, decreasing performance, in addition to adding time to train the model. Further, the longer to train normally means better accuracy, albeit it can be problematic if you have a smaller dataset or require faster deployment or inference times (38) developed a hybrid approach for deep CNN based on Dolphin-SCA that combines fuzzy deformable fusion along with Dolphin Echolocation-based Sine Cosine Algorithm (Dolphin SCA) as well as Linear Discriminant Analysis (LDA). The model achieved a promising accuracy of 96.3% with their hybrid approach. However, careful consideration is required in any application of this technique as optimization algorithms like SCA increase the computational burden (overhead) that may be unwanted in real-time applications or when dealing with large datasets.

The use of pretrained networks for tumour classification has been investigated a fair amount. For instances, (39) compared a number of deep learning architectures, such as Xception, InceptionV3, ResNet50, VGG16, as well as MobileNet, reporting F1-scores of 97.25–98.75%, with Xception outperforming the other architectures. While the models in this study performed well, there were a number of issues with the study, such as not considering class imbalance which would potentially affect the other classes negatively if they were underrepresented. It should also be noted that there are inefficiencies in training the number of models as there would be classes. Finally, deep learning models learn a very large number of abstract features from the training data, which adds to the computational burden, which is problematic in many clinical scenarios, as practitioners often need to make decisions in real-time (40), they reported on their self-defined ANN and a CNN based model for brain tumour detection. They reported brain tumour detection accuracies were 26% better in training and 14% better in testing when using the CNN compared to the ANN. This comparison shows that CNNs are better suited for complex image-based tasks. Nevertheless, the use of a self-defined ANN would have limited the model because it would not easily incorporate the advanced methodologies such as transfer learning or data augmentation that dominate the field today. In the same vein, (2022) (41) applied Convolutional Dictionary Learning with local constraints and achieved training accuracies between 97.74 and 97.85%; however, given dictionary learning’s reliance in constructing simpler patterns, this may negatively influence the model’s ability to capture the complex spatial features that are necessary for accurate classification of brain tumours.

Díaz-Pernas et al. (42) reported a multi-scale CNN-type methodology which categorized three classes of brain tumours glioma, pituitary, and meningioma creating three CNNs with the same structure, but different layers. This strategy had an average accuracy of 97.3% but had limitations in relation to computational demands, taking around 5 days to analyze 3,064 T1-weighted images. It is unfortunate that the high computational load is crippling, especially when you have a high volume of images/subjects.

The (43) used auto-encoders for noise reduction and feature extraction to introduce deep learning to their comparison with other ML algorithms (e.g., SVM, KNN, Random Forest (RF), Logistic Regression and Stochastic Gradient Descent). CNNs consistently showed a higher level of performance than traditional approaches. However, the added complexity of auto-encoders may limit their applicability in real-time medical scenarios, where quick, reliable results are necessary. Recent advancements in deep learning for brain tumor classification have shown substantial improvements in diagnosis accuracy and efficiency. One notable approach is the Dual-Stream Contrastive Latent Learning GAN (DSCLPGAN), which leverages a dual-stream generator to augment MRI datasets. The model captures both local and global features of MRI images, producing diverse synthetic data that enhances classifier performance, especially in the presence of class imbalances (44). This methodology addresses the limitation of traditional GANs, which struggle with mode collapse and lack diversity in synthetic image generation.

Another significant advancement is the Rotation Invariant Vision Transformer (RViT), which incorporates rotated patch embeddings to enhance the classification accuracy of brain tumors. The introduction of rotation invariance allows the model to handle varying orientations of tumor images, a common challenge in medical imaging. The approach has demonstrated superior performance, achieving a sensitivity of 1.0 and an overall accuracy of 98.6%, outperforming conventional convolutional neural networks (CNNs) in handling global features and spatial dependencies (45).

The Swin Transformer has also emerged as a powerful tool in the domain of medical image analysis. By introducing the Hybrid Shifted Windows Multi-Head Self-Attention (HSW-MSA) module and Residual Multi-Layer Perceptron (ResMLP), the Swin Transformer model significantly enhances brain tumor diagnosis. This model improves classification accuracy while reducing computational complexity and memory usage. The model achieved an impressive accuracy of 99.92%, surpassing previous deep learning-based methods (46). Its success in medical image analysis underscores the potential of transformer architectures in overcoming the limitations of traditional CNN-based approaches.

### 1.1 Objectives of the study

The primary objectives of this study are:

- To propose a hybrid DLmodel that integrates Autoencoders (AE) for feature extraction, Conditional Generative Adversarial Networks (cGAN) for synthetic data generation, and Swin Transformers for the classification of brain tumors.
- To address the challenges of data imbalance and limited annotated datasets by leveraging the synthetic data generated through cGAN, thereby improving the robustness and generalization capability of the model.
- To evaluate the performance of the proposed model on publicly available brain tumor datasets (e.g., Kaggle and Figshare), and demonstrate its ability to achieve superior accuracy and performance compared to state-of-the-art methods in the field of medical image classification.
- To explore the potential for clinical deployment by showing that the hybrid model can handle diverse tumor types and MRI image variations, making it a promising tool for real-world applications in medical diagnosis.

This study puts forth a solid design framework for Big Brain tumour classification using enhanced feature set conditional (cGANs) with a specialized classifier labeled the GAS model using a Swin Transformer structure. The design takes advantage of an autoencoder being trained on real MRI images to learn a compact latent space to classify the images. The GAN discriminator uses the encoder discriminating capabilities to improve on distinguishing real vs. synthetic images compared to generated images alone. The GAN, conditioned on tumour type labels, produces realistic images with complicated construction-wide variations. The re-aligned dataset augmented the real MRI images with the synthetic images to form a vast dataset resolving data scarcity while improving robustness.

The classification part of our work fine-tunes the Swin Transformer, the state of the art architecture that applies shifted window attention to learn local as well as global dependencies in high-resolution images. This architecture is efficient in terms of computation and represents powerful features, which makes it well suited for medical imaging. Training on the augmented dataset greatly improves the model’s generalization properties and shows how generative modeling can be harnessed with attention-based architectures to yield very fast and accurate systems for detecting brain tumours.

The contributions of this work include:

- This work introduces a novel hybrid model combining an AE for feature extraction, a cGAN for synthetic data generation, and a Swin Transformer for classification. This unique combination leverages the strengths of each individual component to enhance the overall performance of the system.
- The research proposes an innovative approach to feature extraction, utilizing an Autoencoder to efficiently capture and represent the essential features of the input data. The AE’s ability to learn compressed latent representations facilitates improved data interpretation and sets the foundation for subsequent processing stages.
- The study contributes by integrating a Conditional GAN within the architecture to generate high-quality synthetic data. By conditioning the generator on the extracted features, the model is able to produce realistic and diverse synthetic data, which enhances the robustness of the overall system, particularly in scenarios with limited real data.
- A key contribution of this work is the adoption of the Swin Transformer for classification. The Swin Transformer, with its hierarchical attention mechanism and efficient processing of high-dimensional data, enables more accurate and scalable classification. This contributes significantly to the model’s ability to handle complex data in a computationally efficient manner.
- The proposed model not only improves the quality of feature extraction and classification but also offers a mechanism for augmenting datasets through synthetic data, which improves the generalization ability and performance of the model in diverse scenarios. In the proposed model, a different number of epochs has been used to reduce the model’s complexity.

## 2 Proposed GAN model

This section describes the complete pipeline proposed for brain tumour classification, integrating data preprocessing, feature learning through an autoencoder, synthetic data generation using a class-conditional GAN, and final classification using a fine-tuned Swin Transformer. Each component is explained with layer-wise architectural details, rationale, and training specifications.

**Figure 1:**
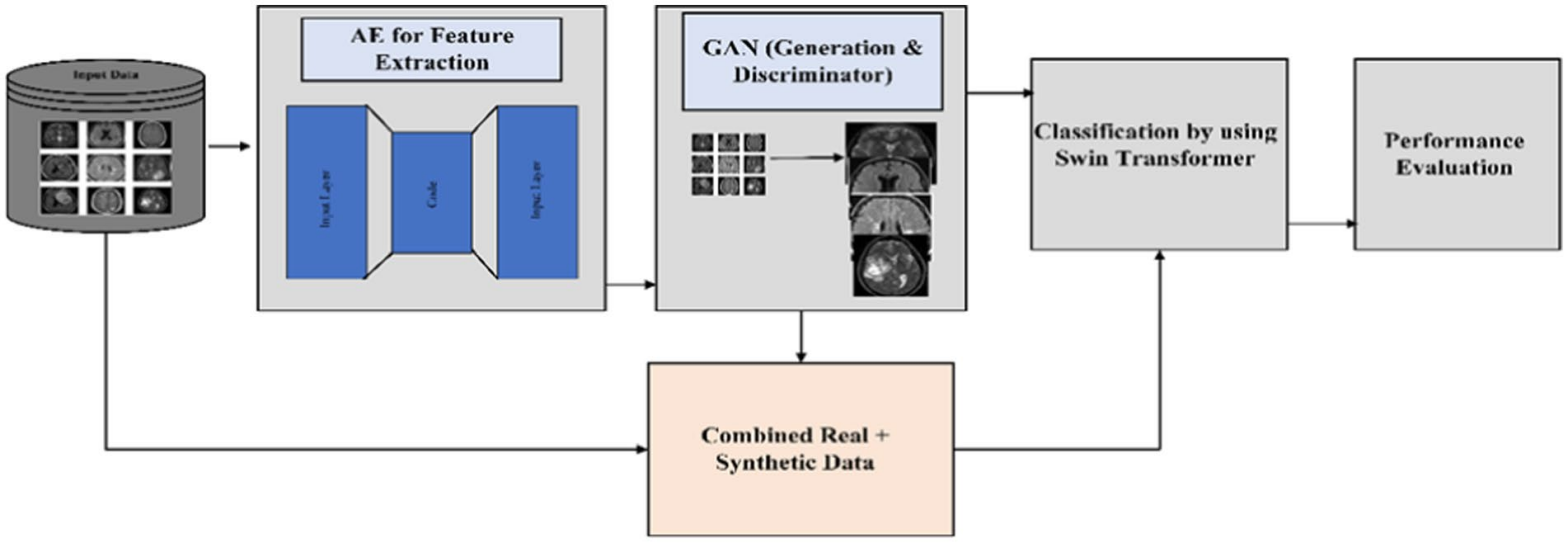
Schematic representation of the proposed architecture flow.

Figure 1 illustrates a hybrid approach using AE for feature extraction and cGAN for data generation and discrimination. The synthetic sample data and real data will be used for classification analysis with a Swin Transformer and performance evaluation.

**Figure 2:**
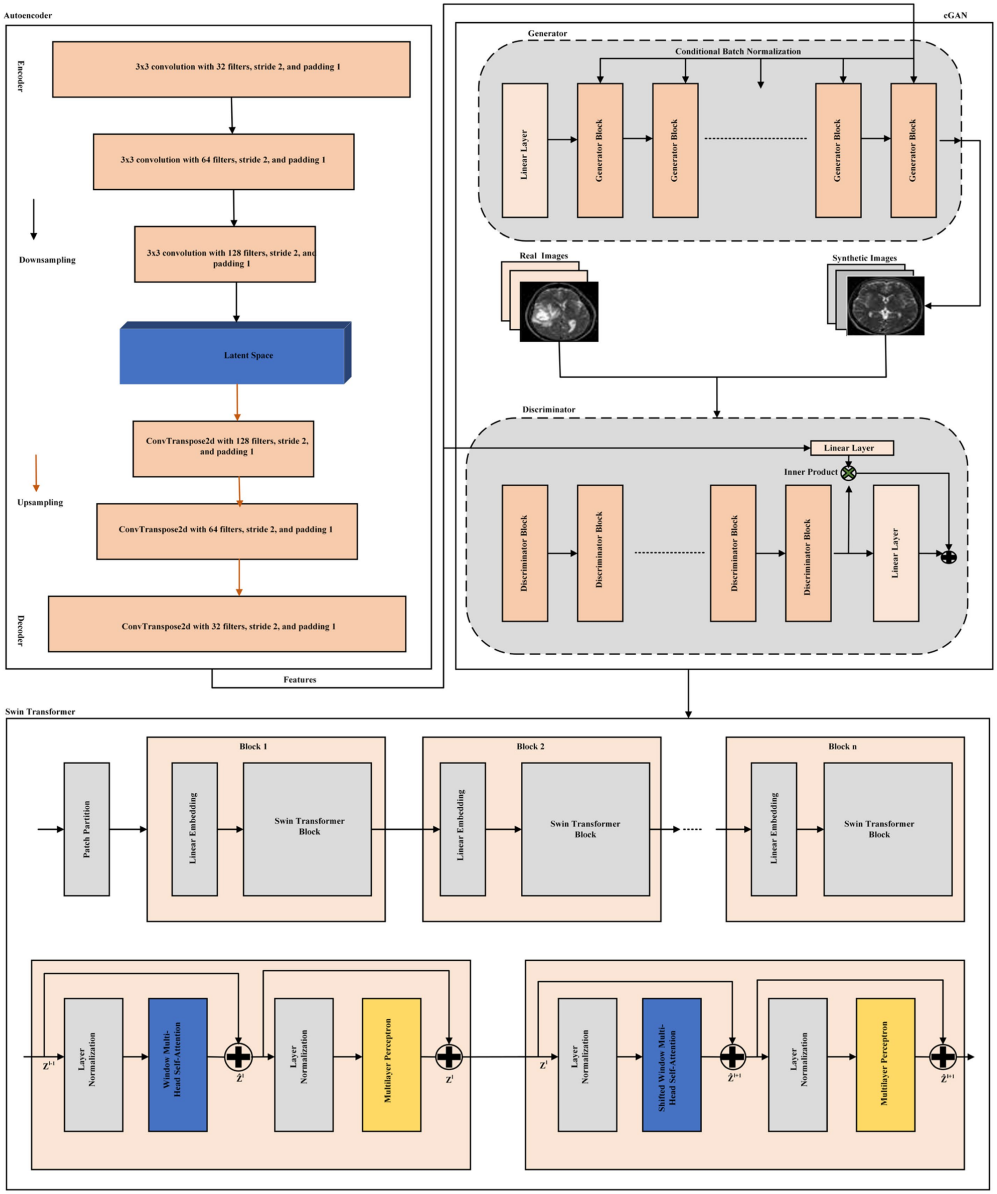
Proposed model architecture integrated an AE for feature extraction, a cGAN for synthetic data generation, and classification using a Swin Transformer.

Figure 2 illustration reflects an integrated approach combining an AE for feature extraction, a Conditional Generative Adversarial Network (cGAN) for synthetic data generation, and classification with a Swin Transformer model. The initial step is the autoencoder where input data is first sent through convolutional layers to downsample and extract a latent space representation. The latent features are then upsampled to reconstruct the image, enabling feature extraction at the next steps.

### 2.1 Preprocessing

The preprocessing pipeline for the MRI images involves organizing the dataset into four tumour classes (Notumour, Glioma, Meningioma, Pituitary) and storing the file paths and labels in a Pandas DataFrame. The dataset is split into training, validation, and test sets using an (80–20)% split while maintaining class balance with the *train_test_split* method. Images are processed with multiple transformations based on the model. The Autoencoder (AE) processes images resized to 64×64, converted to tensors, and normalized to values ranging from [−1, 1]. The Swin transformer processes images are resized to 224×224, and normalized with [0.485, 0.456, 0.406] (mean) and [0.229, 0.224, 0.225] (std) from the pretrained Swin model. Because each model that will be trained requires different input transformations, a custom *ImageDataset* class is declared to load images and apply the proper transformations. In each case, the *DataLoader* objects are treated as batch processes specifically for training data (using the shuffle method) and validation/testing data (no shuffle method).

### 2.2 Autoencoder-assisted feature extraction for discriminator

The Autoencoder’s encoder takes the input images. It converts them into a latent feature space, which the Discriminator utilizes to refine its ability to distinguish real versus fake images. The integration of the Autoencoder’s encoder with the cGAN’s Discriminator contributes to enhanced feature extraction and improved classification performance. The Autoencoder consists of two parts, the encoder and the decoder. The encoder learns to map input images x into a lower-dimensional latent representation z. This can be mathematically expressed as Equation 1:


(1) z=E(x)

where E(x) is the encoder function that processes the input image x and outputs a feature representation z.

after the Autoencoder is trained, the encoder is extracted and frozen to prevent further updates during cGAN training. The Discriminator uses this encoder to extract features from both real images real and fake images G(z, y) generated by the Generator. Let f_encoder_ represent the encoder function, which is used in both Equations 2, 3:


(2) $$f_{\text{encoder}}(x)=E(x)$$

where $f_{\text{encoder}}$ signifies the feature-vector obtained from the encoder of the Autoencoder. These features are then succeeded to the Discriminator to classify whether the image is real (1) or fake (0). The Discriminator’s output is given by:


(3) $$D(x,y)=\sigma (f_{\text{encoder}}(x,y)$$

where σ is the sigmoid activation function, outputting the probability that the image is real.

After the AE process, the cGAN then generates synthetic images to augment the dataset. The encoder component of the Autoencoder enables the Discriminator to extract features from both sets of images, real and fake. The Generator will create a synthetic MRI image from random noise input and the associated class labels. Then, the synthetic image is used to augment the dataset by adding diversity in the dataset. The synthetic images are integrated into the real images to create a dataset of augmented images. This integrated dataset consisting of real images and synthetic images will then be used to train the final Swin Transformer classifier.

### 2.3 Conditional generative adversarial network (cGAN)

A cGAN is used to generate synthetic MRI images for data augmentation. The cGAN consists of two components, the Generator and the Discriminator as present n Equations 4 to 7. The Generator takes random noise *z* and a class label *y* as inputs, producing synthetic images $\hat{x}$ as:


(4) $$\hat{x}=G(z,y)$$

where *G* is the Generator function. The Generator learns to produce images that look like real MRI images. The Discriminator takes an image *x* and the associated label *y*, returning the probability D(x,y) that the image is real:


(5) $$D(x,y)=\sigma (f_{\text{encoder}}(x,y)$$

where σ function, and is the feature extraction function f(x,y)of the Discriminator. The Discriminator returns D(x,y)a probability based on our pretty obvious notation of real (1) vs. fake (0) for the image. Finally, we use binary cross-entropy (BCE) to classify real images as real and images generated by G as fake for the Discriminator loss *L_D_*:


(6) $$\begin{array}{l} L_{G}=\frac{1}{2}(\mathbb{R}x_{\text{real}}\sim p_{\text{data}}[\log D(x_{\text{real}},y)]+{\mathbb{R}}_{z}\sim p_{z}\\ \left[\log(1-D(G(z,y)y))]\right) \end{array}$$

where $x_{\text{real}}$ signifies real images from the dataset, and G(z,y) are the fake images generated by the Generator. The Discriminator is trained to maximize this loss. The Generator loss $L_{G}$ encourages the Generator to produce images that the Discriminator classifies as real:


(7) $$L_{G}={\mathbb{R}}_{z}\sim p_{z}[\log(1-D(G(z,y)y))])$$

**Figure 3:**
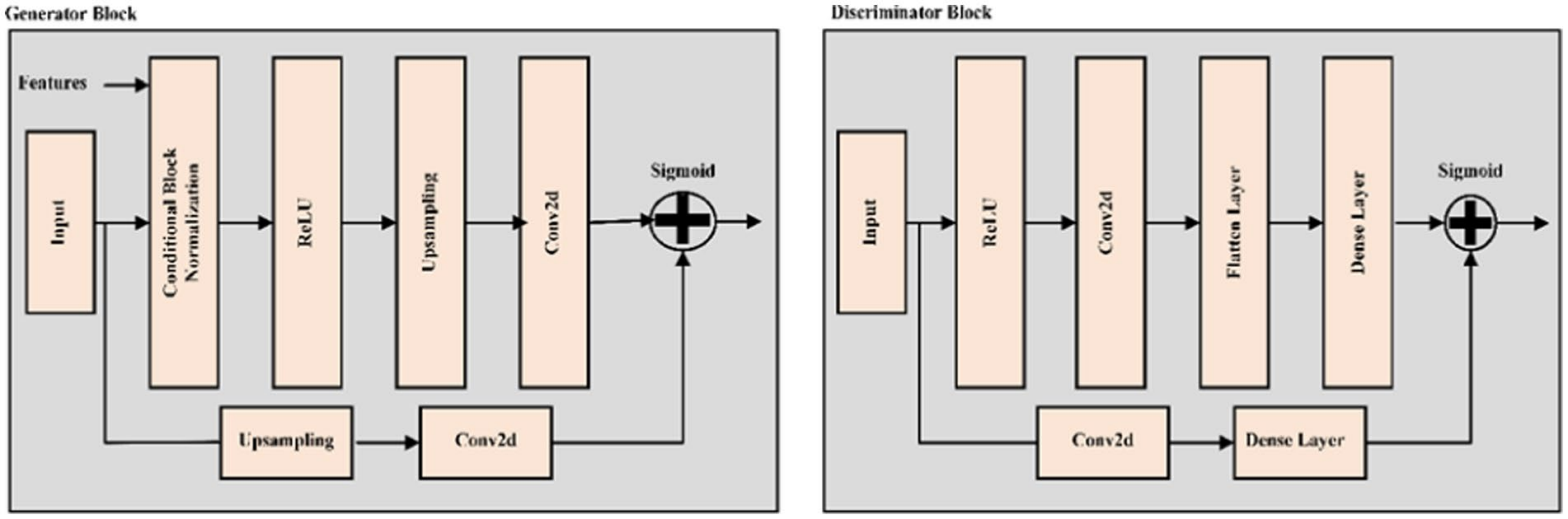
Detailed architecture of generator block and discriminator block.

This loss function ensures the Generator “fools” the Discriminator by producing more realistic images. The Discriminator and Generator are trained alternately, with the Discriminator optimizing $L_{D}\;$and the Generator optimizing $L_{G}$. During training, the Generator learns to create more convincing images, and the Discriminator learns to better differentiate real images from fakes. Figure 3 shows the description of Generator block and Discriminator block.

### 2.4 Swin Transformer for classification

The Swin Transformer model serves as the final classifier for the task of brain tumour classification. The Swin transformer is a vision transformer model that utilizes self-attention mechanisms that learn local and global features in the representation of the images. The model architecture is initialized with a pretrained Swin Transformer from the timm library, a small Swin Transformer known as the Swin-Tiny, and it is fine-tuned for the classification of MRI images into four classes, Notumour, Glioma, Meningioma, and Pituitary. The input image is processed by a Swin transformer backbone to take feature representations from the image. Mathematically this is represented as Equation 8:


(8) $$f_{\text{swin}}(x)=\text{Swin}\_\text{Transformer}(x)$$

where $f_{\text{swin}}(x)\;$is the output feature vector extracted by the Swin Transformer for the input image *x*. The output feature vector is then passed through a fully connected classifier head to predict the class label in Equation 9:


(9) $$y_{\text{pred}}=W_{\mathrm{fc}}f_{\text{swin}}(x)$$

where $y_{\text{pred}}$ is the predicted tumour class, and $W_{\mathrm{fc}}$ represents the weights of the fully connected (FC) layers used for classification. The Swin Transformer is trained using the combined dataset, which includes both real and synthetic MRI images. During training, Cross-Entropy Loss is used as the objective function to compare the predicted class $y_{\text{pred}}\;$with the ground truth labels. The model is optimized to minimize this loss using Equation 10:


(10) $$L_{\mathrm{CE}}=-\sum_{c=1}^{C}y_{C}\log(p_{C})$$

where *C* is the number of classes, $y_{C}$ is the true label (one-hot encoded), and $p_{C}$ is the predicted probability for class *c*.

## 3 Result analysis

### 3.1 Experimental setup

Two publicly available datasets of MRI samples were utilised to validate the proposed GAN model. The following sections describe the datasets, preprocessing, and training configurations used for the experiments.

Dataset I: The Kaggle Brain MRI Dataset includes over 3,000 labeled MRI images, organized into four classes: glioma, meningioma, pituitary tumour, and no tumour. The images were recorded in separate training and testing folders, and in various views and resolutions. Images were collected from a variety of slices: axial, sagittal, as well as coronal. In this study, the dataset was merged and organized into classes using stratified sampling to maintain the proportions of the various classes. The dataset was separated into training, validation, and testing sets; with 80% allocated to training, and 10% to validation, and testing sets, which took place in an 80:10:10 division.

Dataset II: The second data set, called the Figshare Brain tumour Data Set, contains 3,064 (grayscale) MRI scans across three tumour types: glioma (1,426 images), pituitary tumour (930 images), and meningioma (708 images). The images also include different views (axial, coronal, sagittal) and quality and variety. However, the data had no inclusion of a ‘no tumour’ class, as did the Kaggle dataset. The types II data set was randomly split using stratified sampling where 80% was for training, 10% for validation, and 10% for testing.

Hardware and Training Setup Details: All experiments in this study were implemented in Google Colab Pro to utilize the computational resources necessary for training the models. The training setup had an NVIDIA Tesla T4 GPU (16 GB of VRAM, 13 GB of RAM), with a 2-core Intel Xeon virtual machine, all of which was run as a virtualized environment. The hardware was utilized to run the entire training pipeline, including the autoencoder, cGAN, and the classifier. Google Colab Pro enabled the models to be run in the cloud and allowed for the efficient execution of the computationally intensive models. The hyperparameters for the proposed model were selected through empirical tuning, guided by prior work and practical heuristics. The initial values were chosen from standard default settings and adjusted based on training stability, validation performance, and convergence speed. For the autoencoder, the Adam optimizer was used with a learning rate of 1 × 10^−31^, and the Mean Squared Error (MSE) loss function was employed to minimize the difference between the original and reconstructed images. For the cGAN, we used the Adam optimizer with β1 = 0.5 and β2 = 0.999, with a learning rate of 1 × 10^−4^ and the standard cGAN loss to differentiate real and generated images. The Swin Transformer classifier utilized the AdamW optimizer with a learning rate of 3 × 10^−5^, Cross-Entropy Loss for multi-class classification, and the CosineAnnealingLR scheduler to decay the learning rate throughout training. As for data augmentation, synthetic images were generated using the cGAN to alleviate data imbalance. For Dataset I, we generated 500 synthetic images per class, totaling 2,000 synthetic images across the four classes (Notumor, Glioma, Meningioma, Pituitary). Similarly, for Dataset II, 500 synthetic images were generated per class, resulting in 1,500 synthetic images for the three classes (Glioma, Meningioma, Pituitary). These synthetic images augmented the original datasets, improving the model’s generalization ability and mitigating the challenges posed by class imbalance.

Training Time: The training time for each component of the model was as follows: The autoencoder took approximately 215 s to train for 15 epochs, with an average epoch time of about 14.3 s. It was trained once on the full training data. The GAN, comprising both the Generator and Discriminator, required around 8,200 s to complete 100 epochs, with an average epoch time of 82 s. It was trained once on the entire dataset. The classifier, which was trained independently for each of the three folds, took approximately 1,831 s per fold for 30 epochs, with an average epoch time of 61.1 s, with a variation of ± 0.25 s. In total, the classifier’s training time across all three folds amounted to about 5,493 s (91.5 min), aggregating the time across the 3-fold cross-validation process.

### 3.2 Performance evaluation using Dataset I

**Figure 4:**
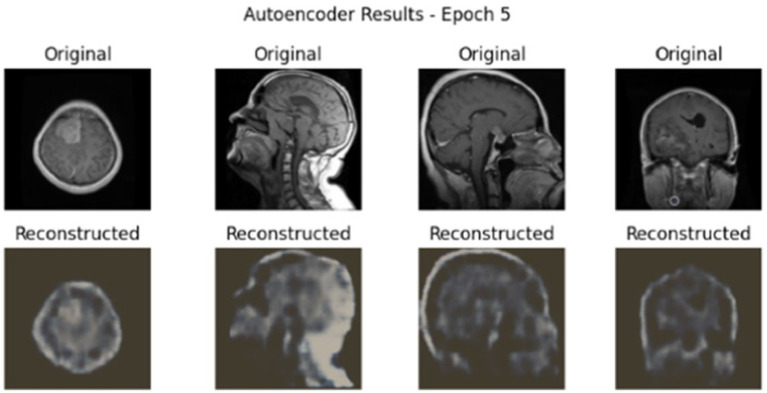
AE reconstruction results for MRI brain scans at epoch 5 on Dataset I.

The proposed GAS model is a robust classifier on the Dataset I, which is classified into four classes: glioma, meningioma, pituitary tumour, and no tumour. In the first step without cGAN, AE and Swin Transformer is applied on data. At each step, the model is trained on different epochs based on performance and to reduce the time complexity. Figure 4 shows the results of first five epochs.

**Figure 5:**
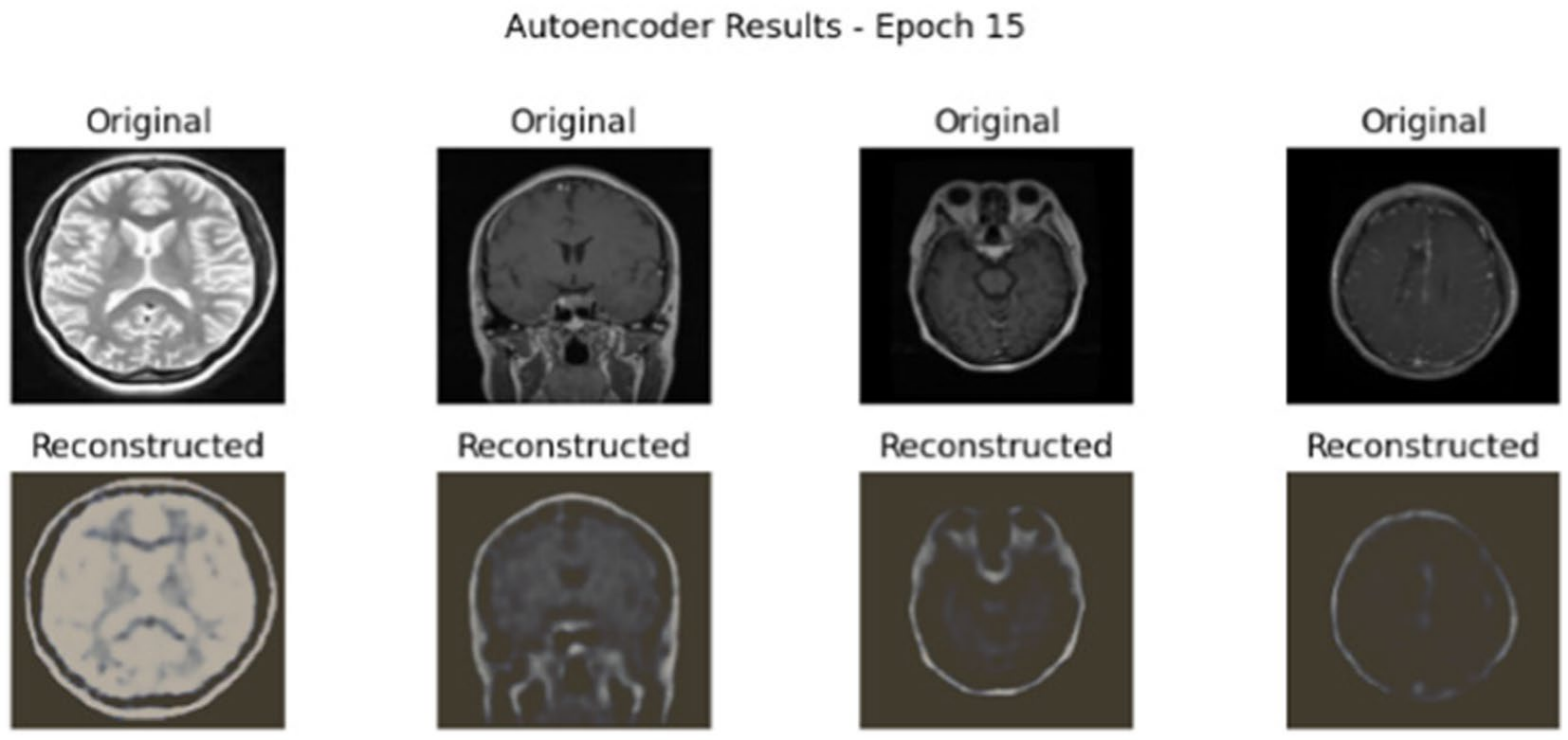
AE reconstruction results for MRI brain scans at epoch 15 on Dataset I.

Figure 5 showcases the performance of an autoencoder model after 15 epochs of training, illustrating the results of reconstructing MRI brain scans. The figure is organized into four areas that each compare the ‘Original’ image to the ‘Reconstructed’ image.

After training of AE, Swin Transformer was used for classification. In this step, a hybrid model of AE and Swin Transformer has been applied to measure the performance of the model without cGAN.

**Figure 6:**
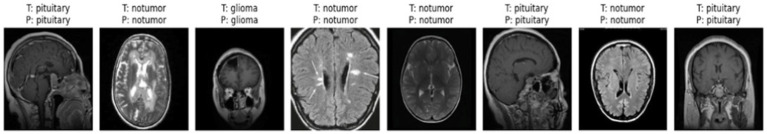
Tumor classification results using the AE + Swin Transformer model.

Figure 6 shows the MRI images classified with the AE + Swin Transformer model. The true label (T) and predicted label (P) are shown below each image. The model classifies a variety of tumours, including pituitary, glioma and nontumour, showcasing the different conditions it was able to classify correctly. The true and predicted labels match up with our accurate model. Additionally, Table 1 shows the performance of the hybrid model of AE + Swin Transformer.

**Table 1:** Performance of hybrid model of AE + Swin Transformer on Dataset I.

Class	Pre (%)	Rec (%)	F1-S (%)
Notumour	100.00	100.00	100.00
Glioma	99.32	97.33	98.32
Meningioma	97.41	98.37	97.89
Pituitary	98.68	99.67	99.17
Accuracy			98.93
Macro avg	98.85	98.84	98.84
Weighted avg	98.94	98.93	98.93

The Table 1 shows that the classification model performs across four tumour types and ``nontumour” instances using Precision(Pre), Recall(Rec), and F1-Score (F1-S) as metrics of interest. The model performs beautifully, having perfect detection of ``nontumour” instances, as well as very good detection of all tumours. The overall accuracy is 98.93%, and the macro and weighted averages also indicate no classes are underperforming. The model is especially good at detecting pituitary tumours with the highest recall, while slightly higher recall or precision varies among tumour types.

**Figure 7:**
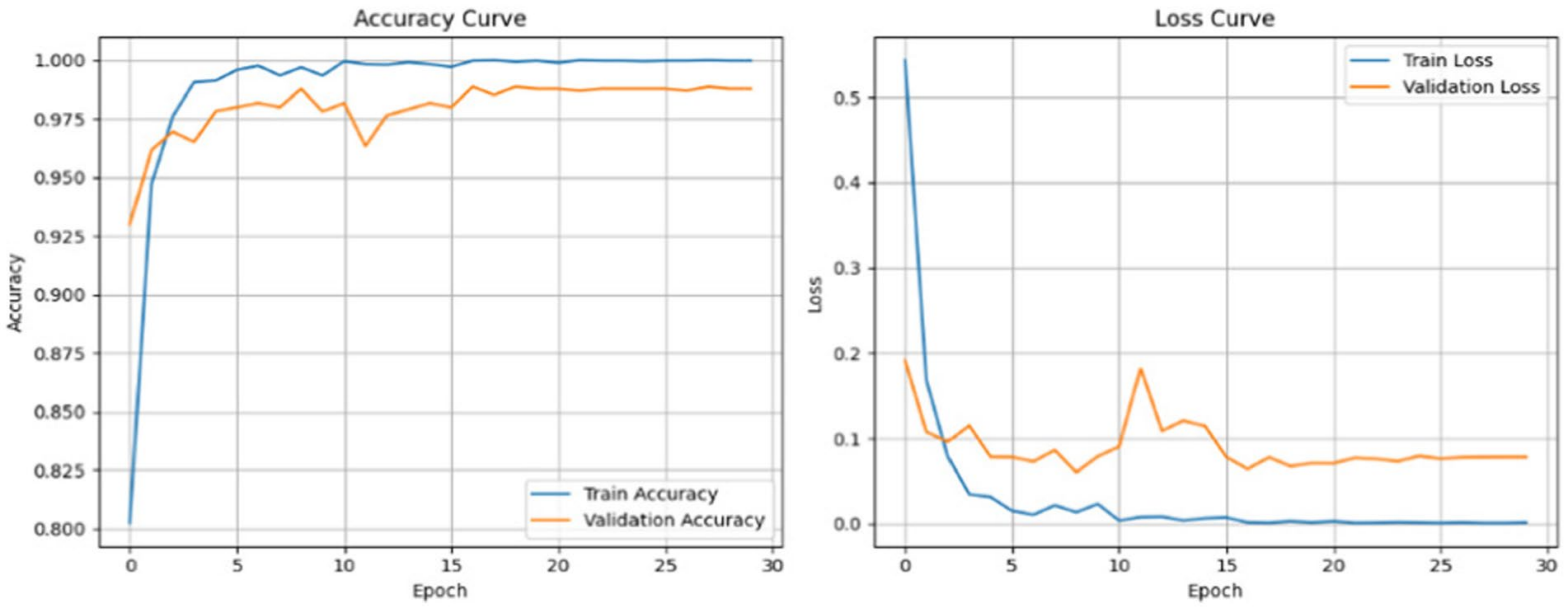
Accuracy and loss curves for trainingand validation over 30 epochs of AE + Swin Transformer.

**Figure 8:**
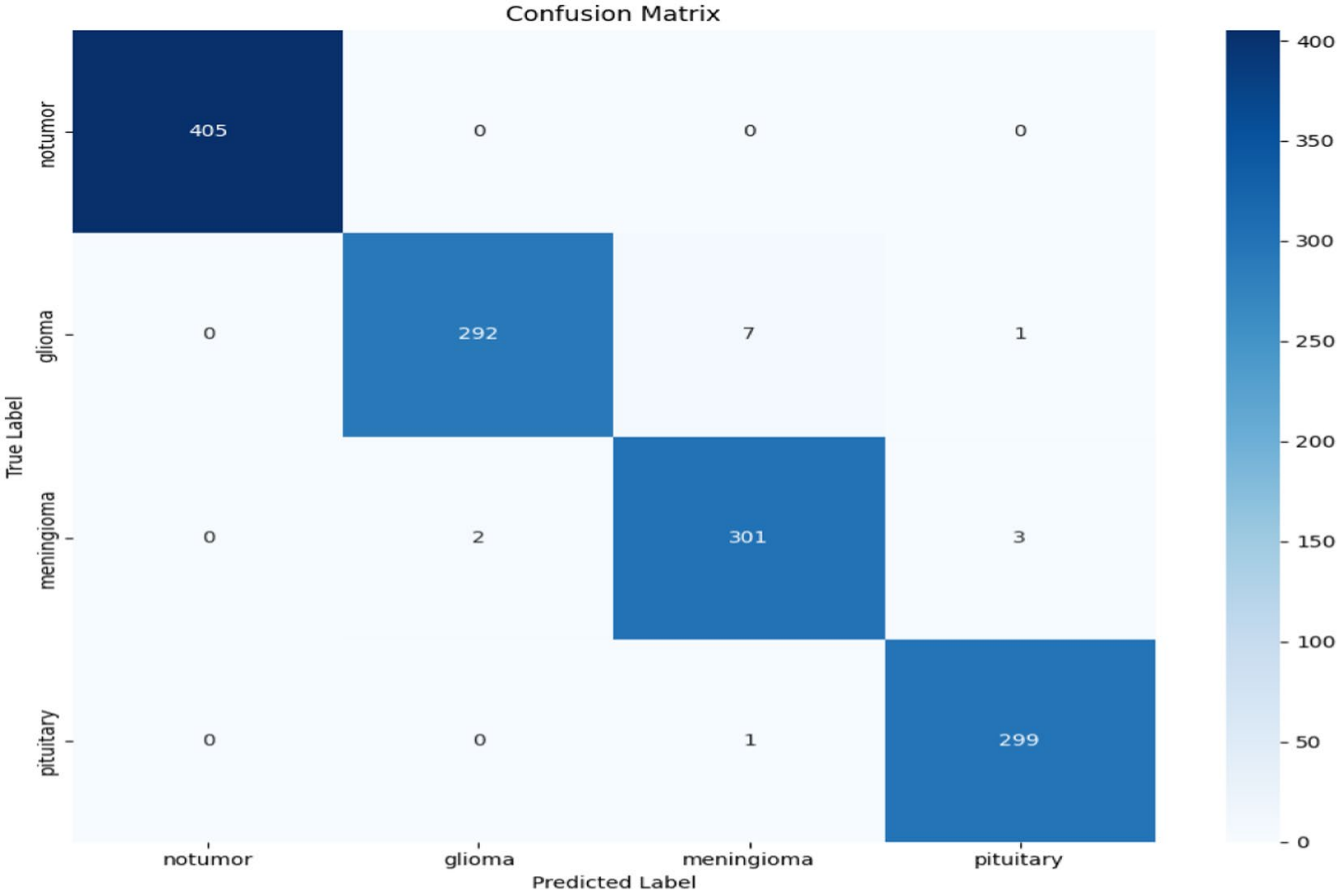
Confusion matrix of AE + Swin Transformer.

In Figure 7, both the accuracy graphs indicate rapid increases in training and validation and the model seems to be close to achieving perfect accuracy. The loss graph indicates a major decrease in loss during initial epochs; the graph plateaus after. The classification performance of the model is shown in Figure 8.

The confusion matrices indicate specific misclassifications, in particular between tumor types, like glioma and meningioma. There are reasons for these misclassifications. First, the visual similarities in the MR images between gliomas and meningiomas, particularly early on, is problematic. Specifically, both tumor types can appear as well-defined, and are often in the same regions of the brain (e.g., cerebral hemispheres), making it difficult for the model to determine the exact type. Furthermore, the morphological characteristics of these two tumors are like shapes, size, textures, etc. Moreover, the small differences between these tumors are occasionally hard for the model to decompose (especially when there are small deviations in training data), and any variances in imaging protocols and scanner protocols for images would also introduce deviations in MRI characteristics. Second, there is a data imbalance, which is exhibited in the confusion matrix. Using the most clinically-representative diagnosis has limits, typically related to less representative cancer instances or situations where a slight bias does not lead to overt misdiagnosis. Third, it is important to understand that tumor classification must only be representative to the images input to the model, particularly based on image qualities and pixels associated with each of the diagnosis categories, or tumor class/diagnosis. It is worth noting that the model is still noticeably high overall accuracy.

**Figure 9:**
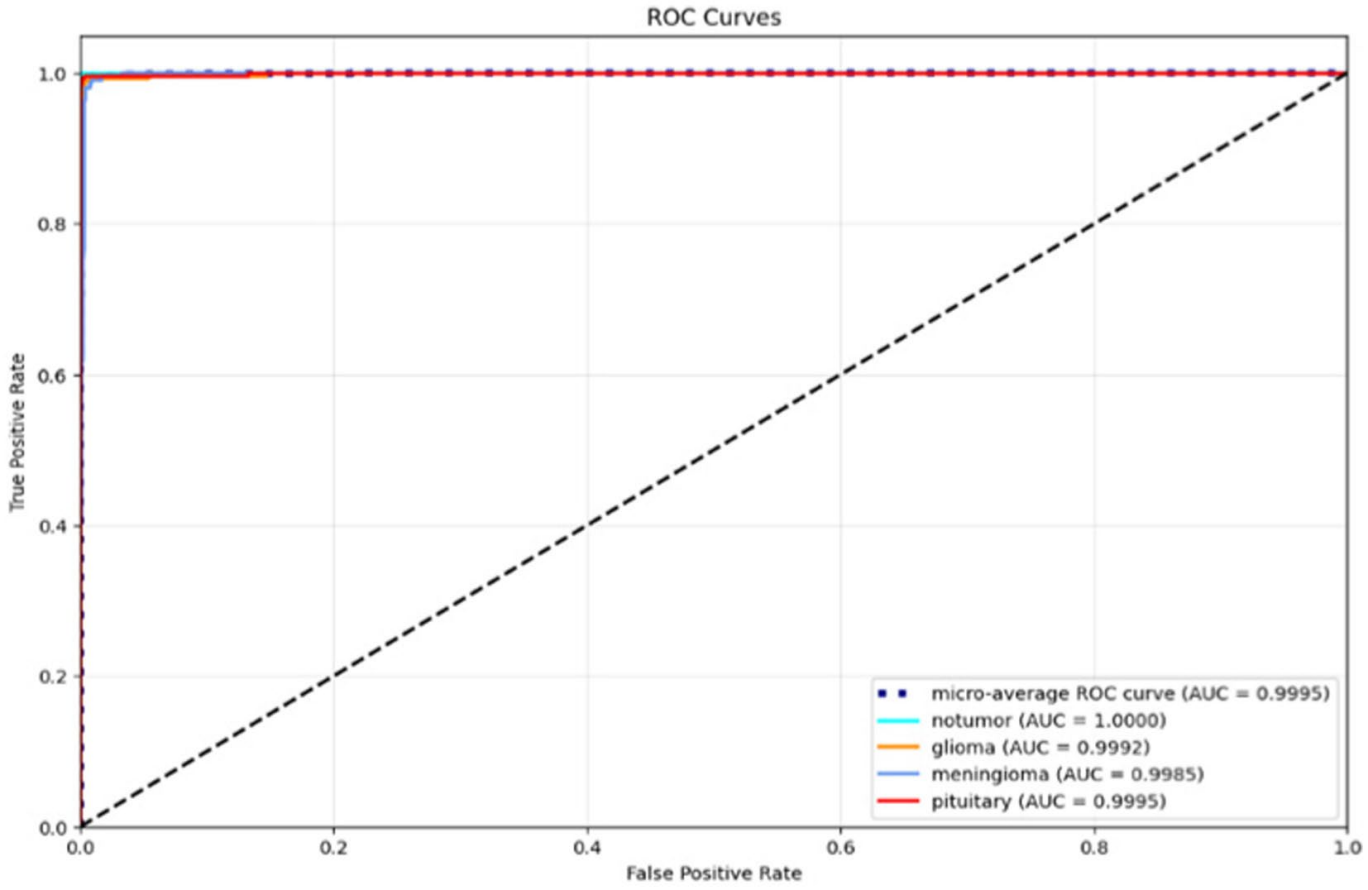
ROC curves of AE + Swin Transformer for tumor detection with AUC values for each class.

The ROC curve in Figure 9 provides strong performance across all classes with high AUC values signifying that the model is very good at teasing apart the different tumour labels and ``nontumour” cases. The micro-average ROC curve exemplifies almost perfect classification performance.

**Figure 10:**
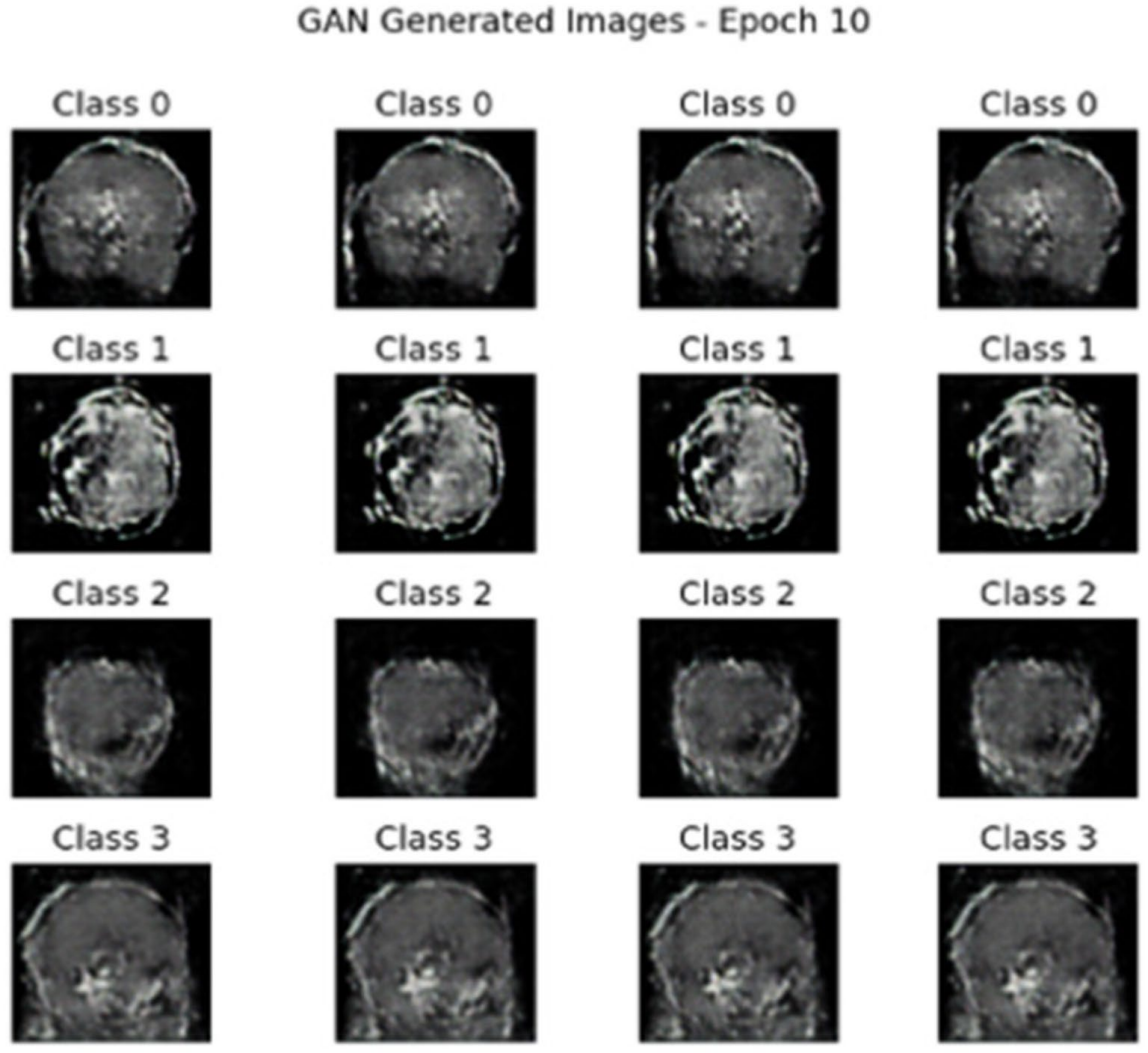
Synthetic images generated by the cGAN at 10 epochs.

**Figure 11:**
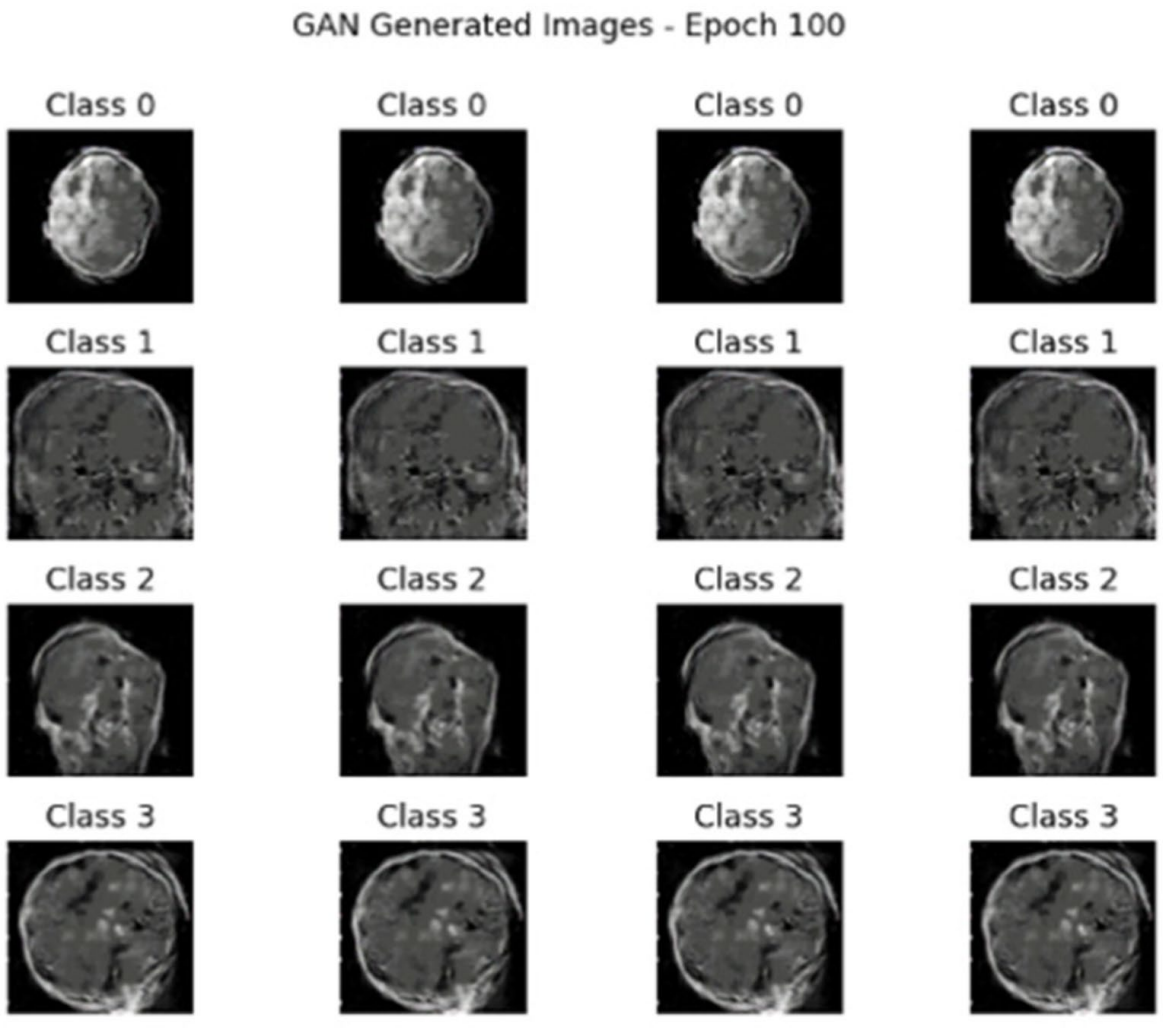
Synthetic images generated by the cGAN at 100 epochs.

After applying the hybrid model of AE + Swin Transformer, cGAN was used to generate synthetic data. Figures 10, 11 illustrate the generated data produced by the cGAN model.

**Figure 12:**
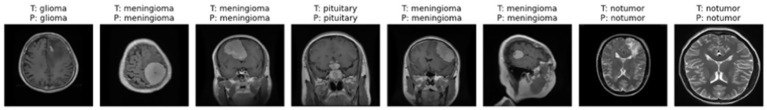
Tumor classification results using the cGAN and AE + Swin Transformer model.

The model was trained using both real images and synthetic images generated from the GAN, with a very successful established accuracy of overall 99.54%. The use of the hierarchical attention layer of the Swin Transformer enabled the GAS model to establish that even seemingly similar tumour types could be distinguished from one another. Figure 12 shows the classification results of cGAN and AE + Swin Transformer.

Table 2 provides class-based evaluation measures: precision, recall, F1-score, and AUC. All classes had model evaluation measures that were extremely high, especially glioma and pituitary tumour, which rated over 99.53% for most measures. The meningioma class had a slightly lower recall rate, which indicated some false negatives, but overall, 99.53% performance.

**Table 2:** Performance of proposed GAS model on Dataset I.

Class	Pre (%)	Rec (%)	F1-S (%)
Nontumour	99.75	99.75	99.75
Glioma	100.00	99.33	99.67
Meningioma	98.70	99.35	99.02
Pituitary	99.67	99.67	99.67
Accuracy			99.54
Macro avg	99.53	99.52	99.53
Weighted avg	99.54	99.54	99.54

**Figure 13:**
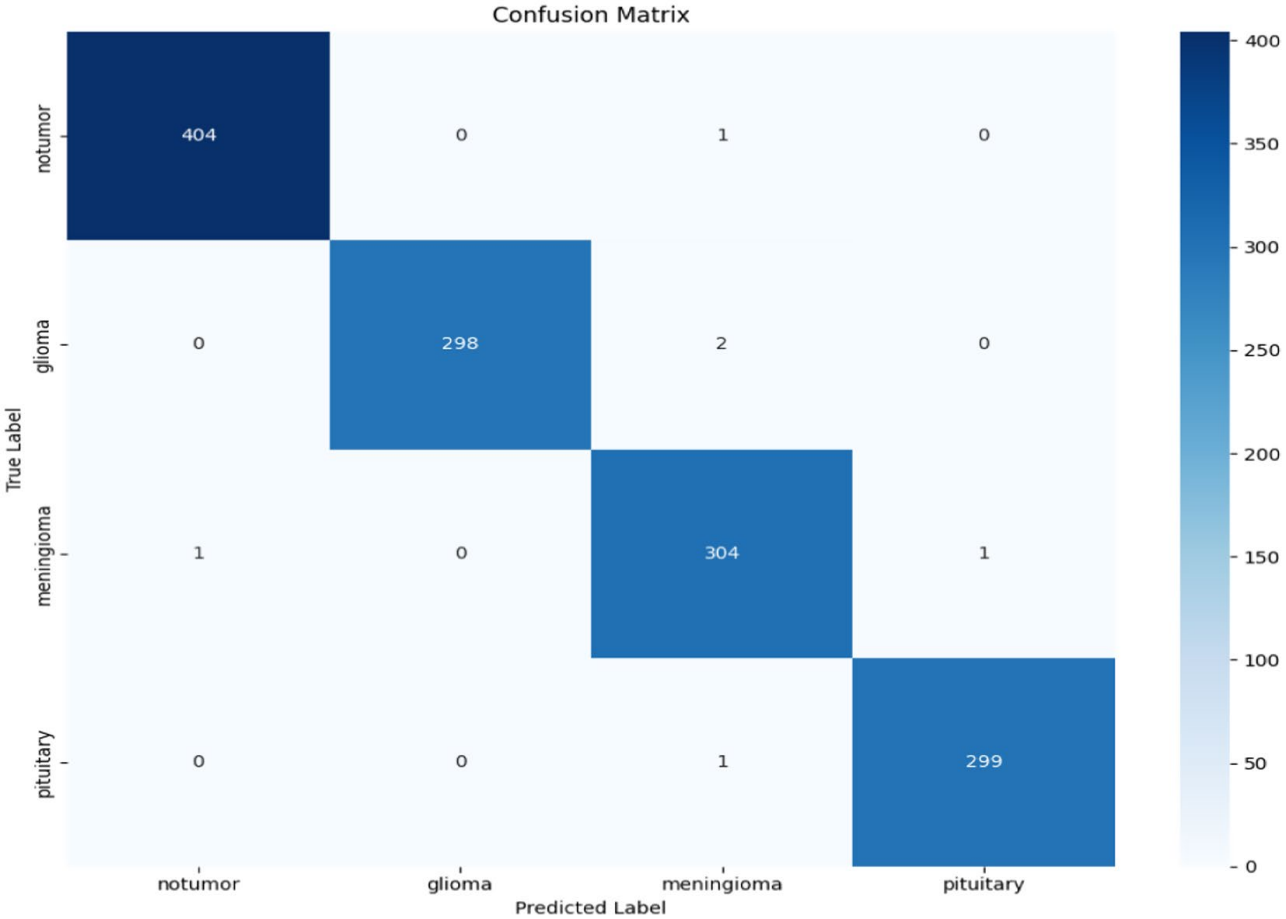
Confusion matrix for the hybrid cGAN and AE + Swin Transformer model.

To better understand the model’s classification capabilities, a confusion matrix was created from the predictions made on the Dataset I test set, shown in Figure 13. The vast majority of the samples were correctly classified and had very few misclassification points. Most of the errors were between meningioma and glioma, tumours that can have overlapping imaging features. However, overall, the confusion matrix highlights the model’s ability to differentiate between all three tumour types with little confusion.

**Figure 14:**
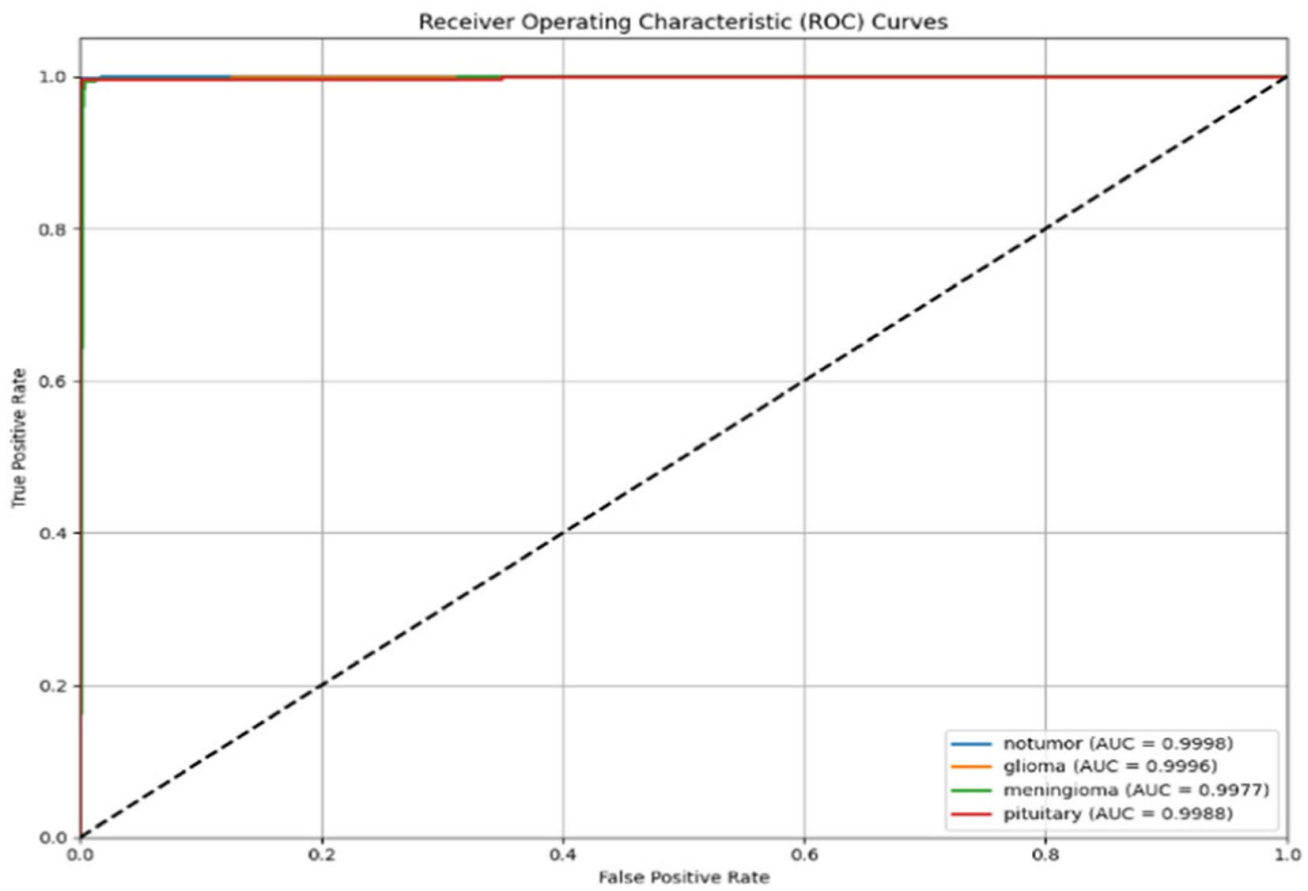
ROC curves for glioma, meningioma, and pituitary classes.

The ROC curves for each tumour class are shown in Figure 14. The ROC curves demonstrate the trade-off between true positive and false positive rates. The AUC was greater than 0.998 for all three classes, indicating the model had strong discriminatory ability. The steep slope of the ROC curves and their proximity to the top-left corner of the plot also provided reassurance that this model would provide the best possible outcomes in clinical practice, where both sensitivity and specificity must be maximized.

**Figure 15:**
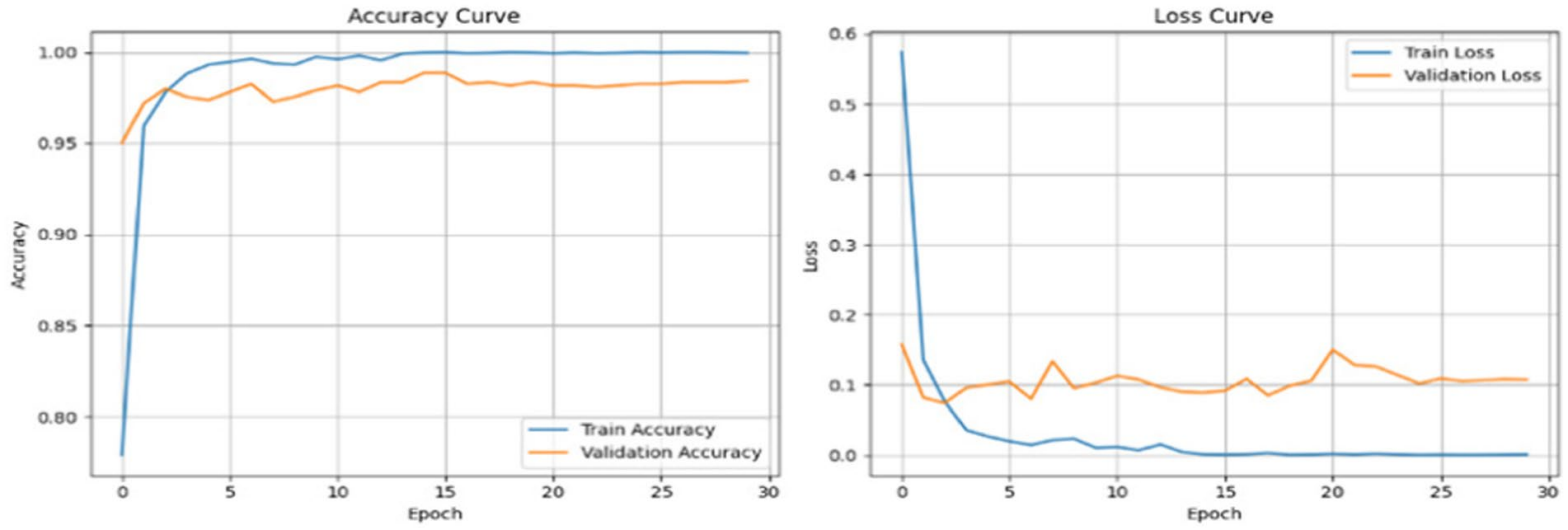
Training vs.validation accuracy and loss curves of Hybrid cGAN andAE+Swin Transformer model for Dataset I.

Figure 15 shows the training dynamics of the model by showing a graph of the training and validation accuracy curve and the training and validation loss curve for 30 epochs. The training and validation curves converge smoothly, and the validation accuracy curves closely follow the training accuracy curves throughout training. No significant overfitting observed, and the validation loss becomes stable when the validation accuracy starts becoming stable. As a consequence, the model has shown to generalize well across the dataset, which is attributed to the inclusion of synthetic data that promoted robust learning. As depicted in Figure 15, the accuracy and loss curves for both training and validation sets are illustrated over 30 epochs for Dataset I. The accuracy curve demonstrated rapid improvement with both the training and validation accuracy stabilizing near 1.0, indicating effective learning. The loss curve showed a rapid loss decrease in the beginning stages of training before stabilizing, showing that the model converged appropriately during training.

### 3.3 Performance evaluation using Dataset II

**Figure 16:**
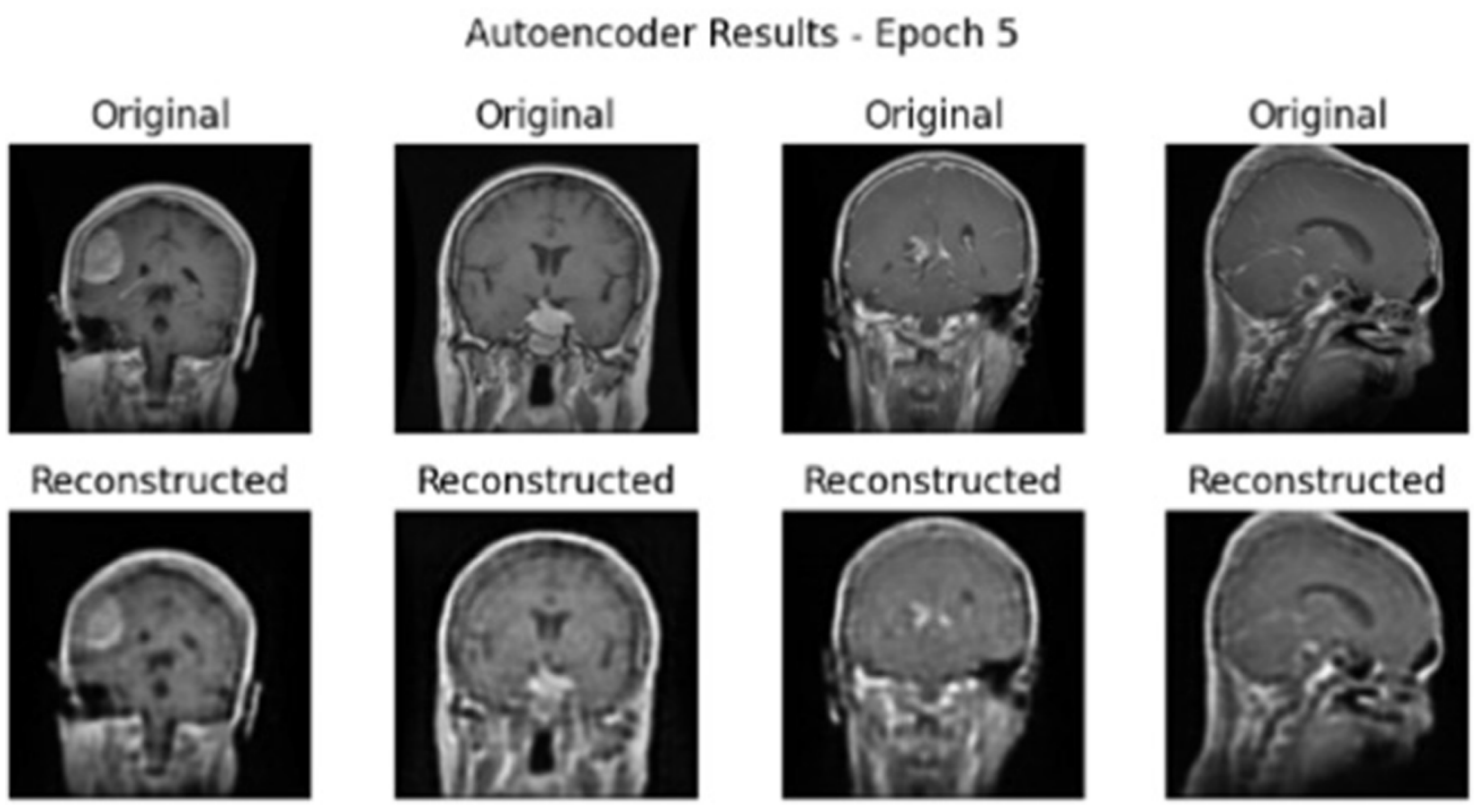
AE reconstruction results for mri brain scans at epoch 5 on Dataset II.

**Figure 17:**
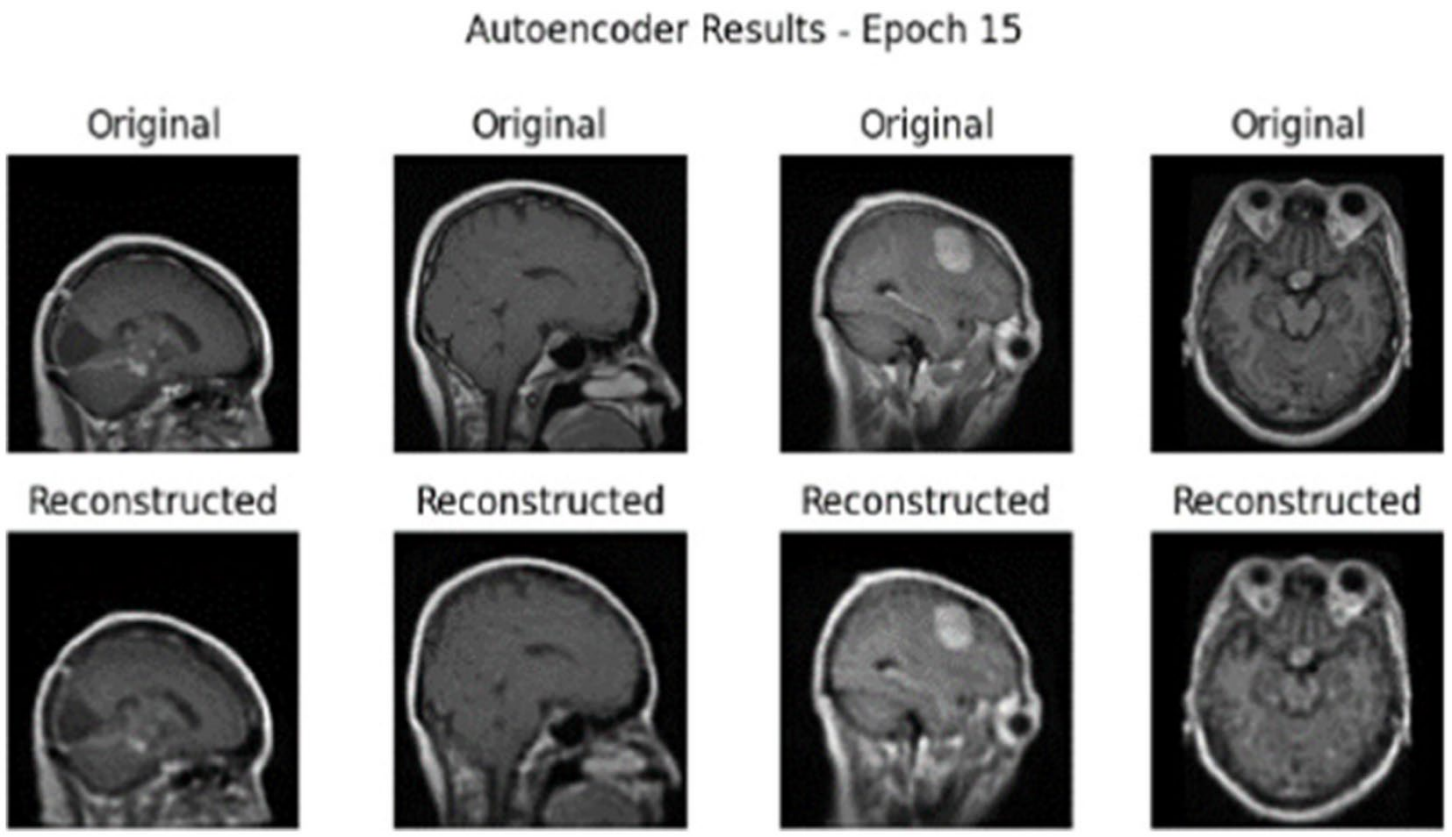
AE reconstruction results for MRI brain scans at epoch 15 on Dataset II.

The GAS model was also tested on a Dataset II consisting of Brain tumour MRIs. The data consisted of three classes of tumours: glioma, meningioma, and pituitary tumour. Experiments were repeated with Dataset II. The first step is to perform the experiment without cGAN. Figures 16, 17 show the results of AE on different epochs.

Table 3 summarizes the overall performance achieved by the hybrid AE + Swin Transformer model on Dataset II, including precision, recall, and F1-score for each class. It is apparent that the model performed quite well with a precision score of 88.7% for glioma, which is very good, and a perfect precision value of 100% for pituitary. Additionally, the overall accuracy for the model was 93.8%. The macro and weighted average values also reflect consistent performance through all classes.

**Table 3:** Performance of hybrid model of AE + Swin Transformer on Dataset II.

Class	Pre (%)	Rec (%)	F1-S (%)
Glioma	88.7	99.3	93.7
Meningioma	98.5	91.5	94.9
Pituitary	100.0	87.1	93.1
Accuracy			93.8
Macro avg	95.7	92.6	93.9
Weighted avg	94.4	93.8	93.8

**Figure 18:**
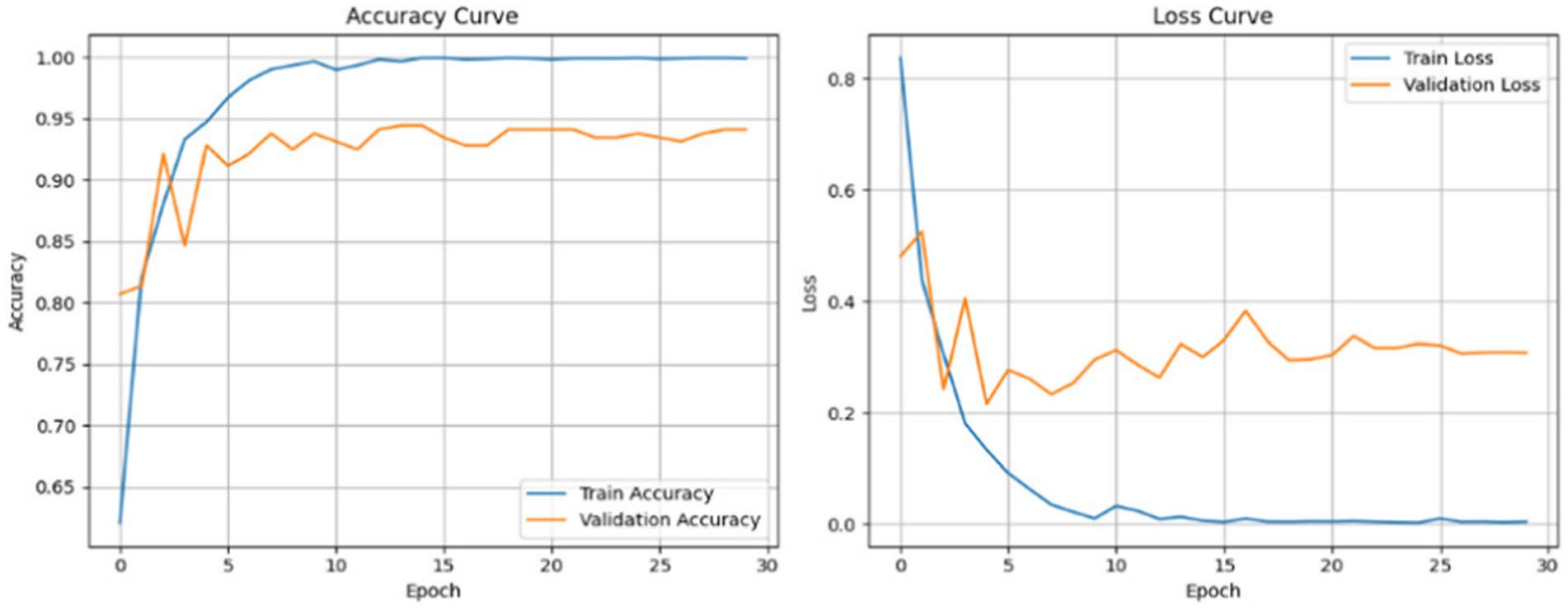
Training and validation accuracy and loss curvesover 30 epochs on Dataset II.

To demonstrate how the model learns, the training and validation accuracy and loss curves through 30 epochs are displayed in Figure 18, demonstrating that the model converges and improves over the course of training.

**Figure 19:**
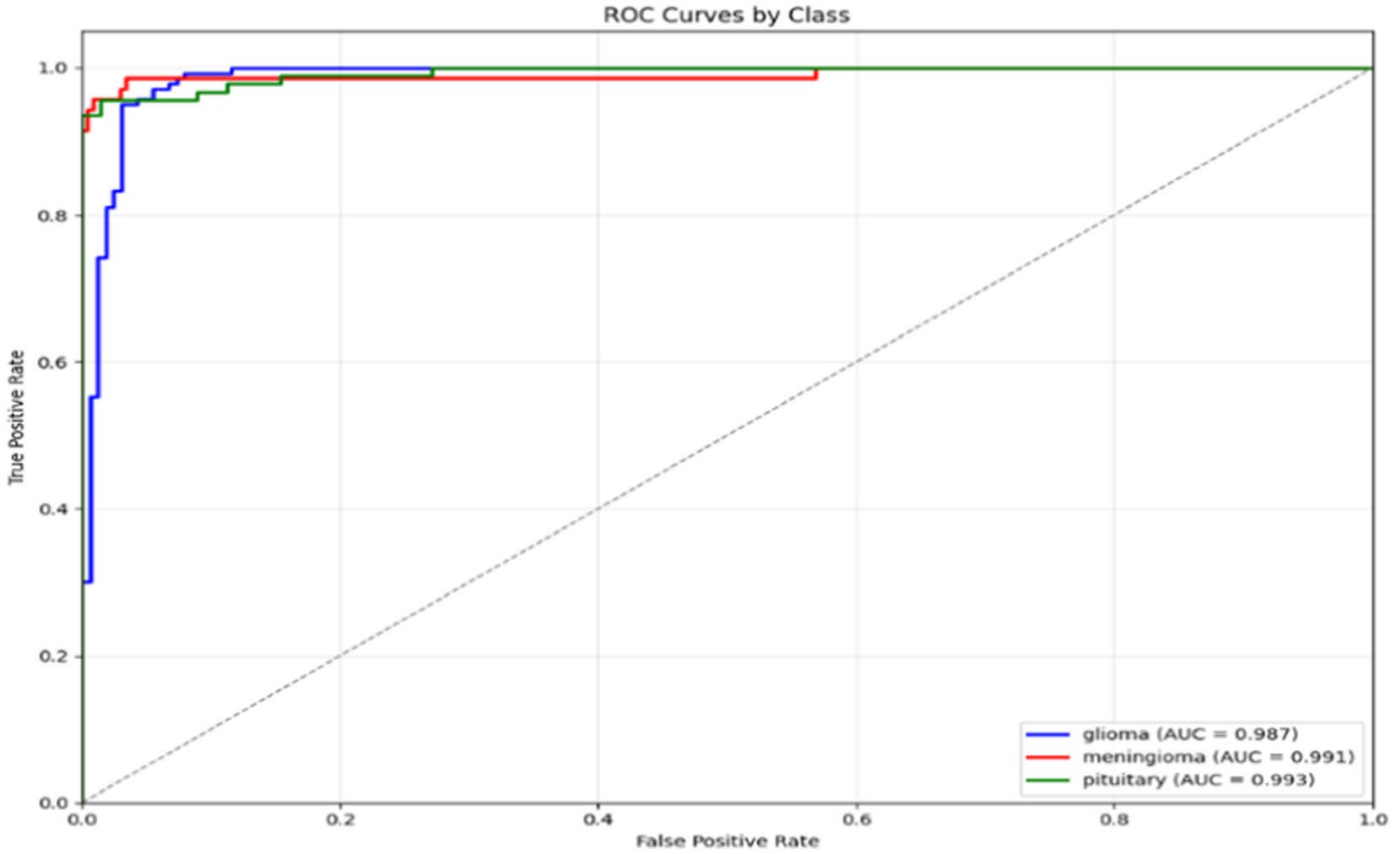
ROC curves of AE + Swin Transformer for tumor detection with AUC values for each class on Dataset II.

After the training process, the model’s ability to distinguish between classes is assessed using the ROC curve. Figure 19 presents the ROC curves, which characterize the model’s performance across all tumour types, including AUCs for each class.

**Figure 20:**
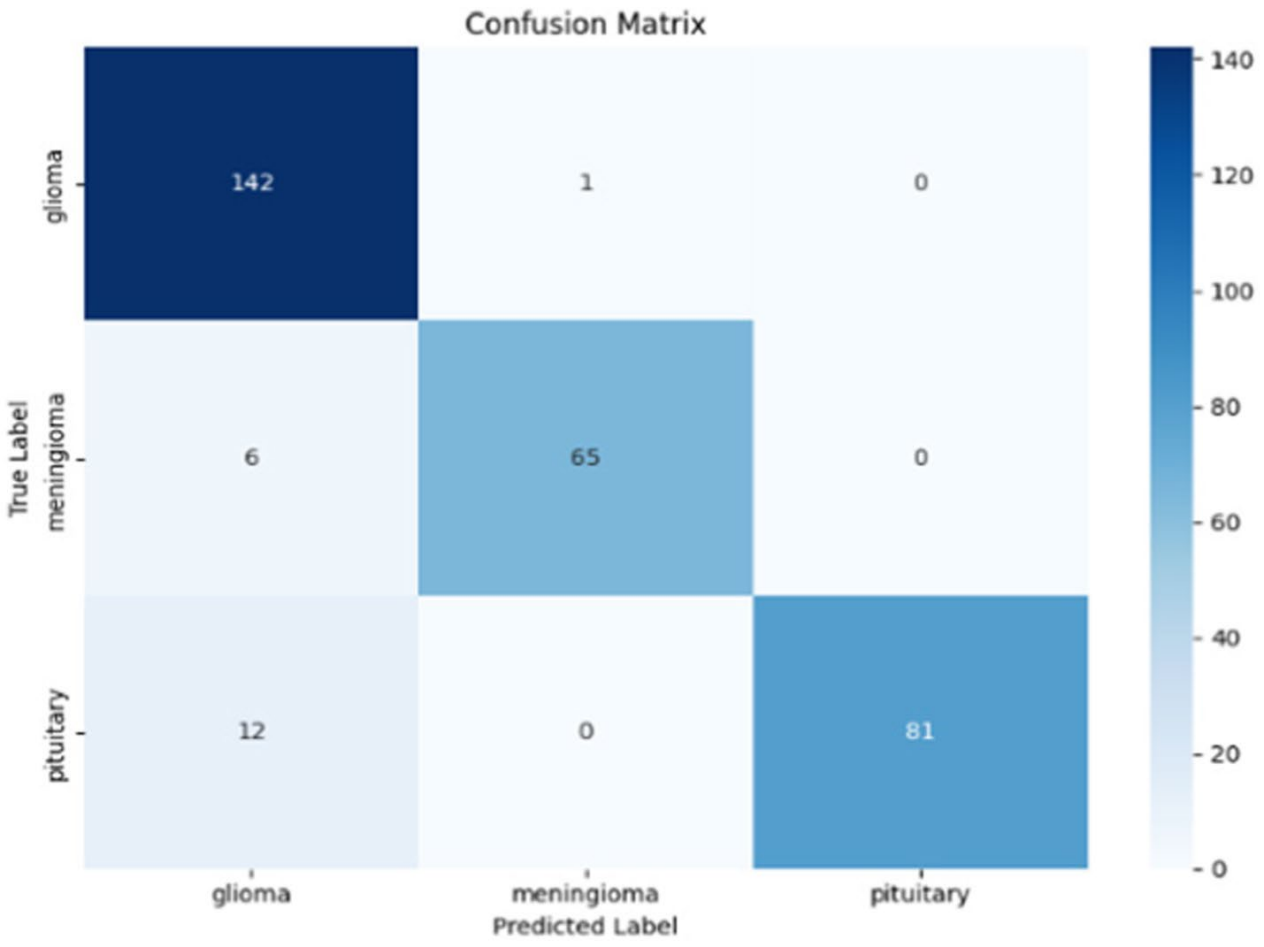
Confusion matrix of AE + Swin Transformer on Dataset II.

While the ROC curve provides an overview of the model’s classification performance, the confusion matrix illustrated in Figure 20 provides a more detailed summary of the model’s predictions, uncovering the true positives, false positives, true negatives, and false negatives for each class.

**Figure 21:**
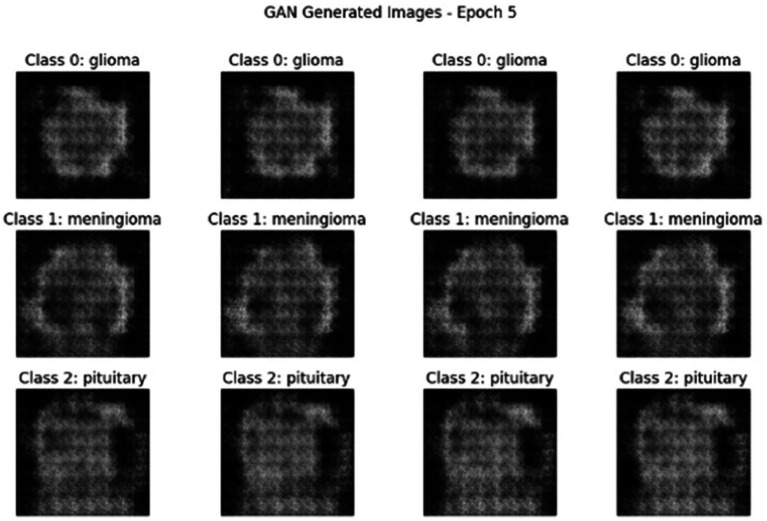
Synthetic images generated by the cGAN at 5 epochs.

**Figure 22:**
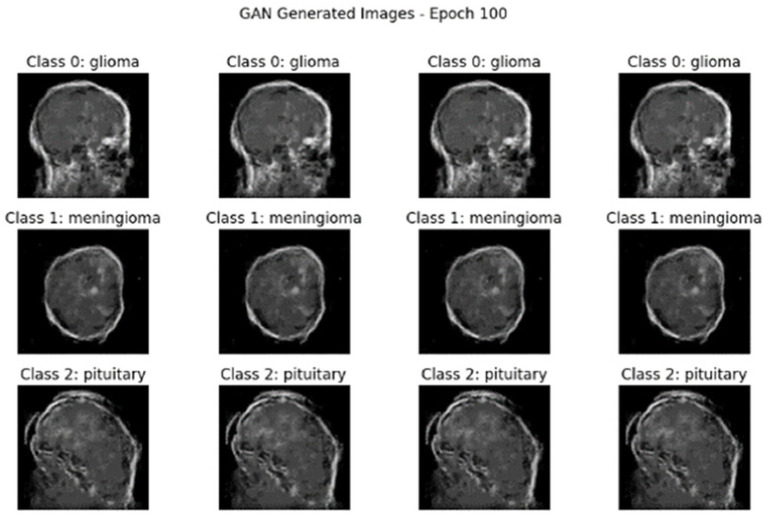
Synthetic images generated by the cGAN at 100 epochs.

After the hybrid model of AE + Swin Transformer, synthetic data has been generated using cGAN with Dataset II. Figures 21, 22 show the results of cGAN.

The results were exceptional as the model achieved an impressive overall accuracy of 98.9%. This shows the model can classify between tumours, along with being able to identify healthy brain MRIs with high confidence. In Table 4, all three classes achieved high values for precision, recall, F1-score, and near-perfect AUC values. The fact that the ``no tumour” class achieved a 100% recall is also important, as it is critical to avoid false negatives in healthy patients.

**Table 4:** Performance of proposed GAS model on Dataset II.

Class	Pre (%)	Rec (%)	F1-S (%)	Support
Glioma	99.3	99.0	99.2	300
Meningioma	98.4	98.4	98.4	306
Pituitary	99.0	99.3	99.2	300
Accuracy			98.9	906
Macro avg	98.9	98.9	98.9	906
Weighted avg	98.9	98.9	98.9	906

Results of GAS model performance by tumour type (precision, recall, and F1-score) on Dataset II, global accuracy of 98.9% are shown in Table 4. The model also demonstrates impressive precision and recall for all tumour types, particularly glioma, meningioma, and pituitary.

**Figure 23:**
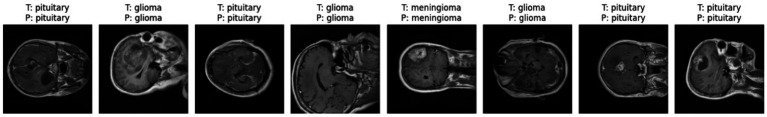
Tumor classification results using the proposed GAS model on Dataset II.

To show this performance, Figure 23 presents MRI images classified by the GAS model, along with true (T) and predicted (P) labels for each tumour type. The images demonstrate the ability of the model to classify discrete tumour types, which are reflected in the impressive metrics in Table 4.

**Figure 24:**
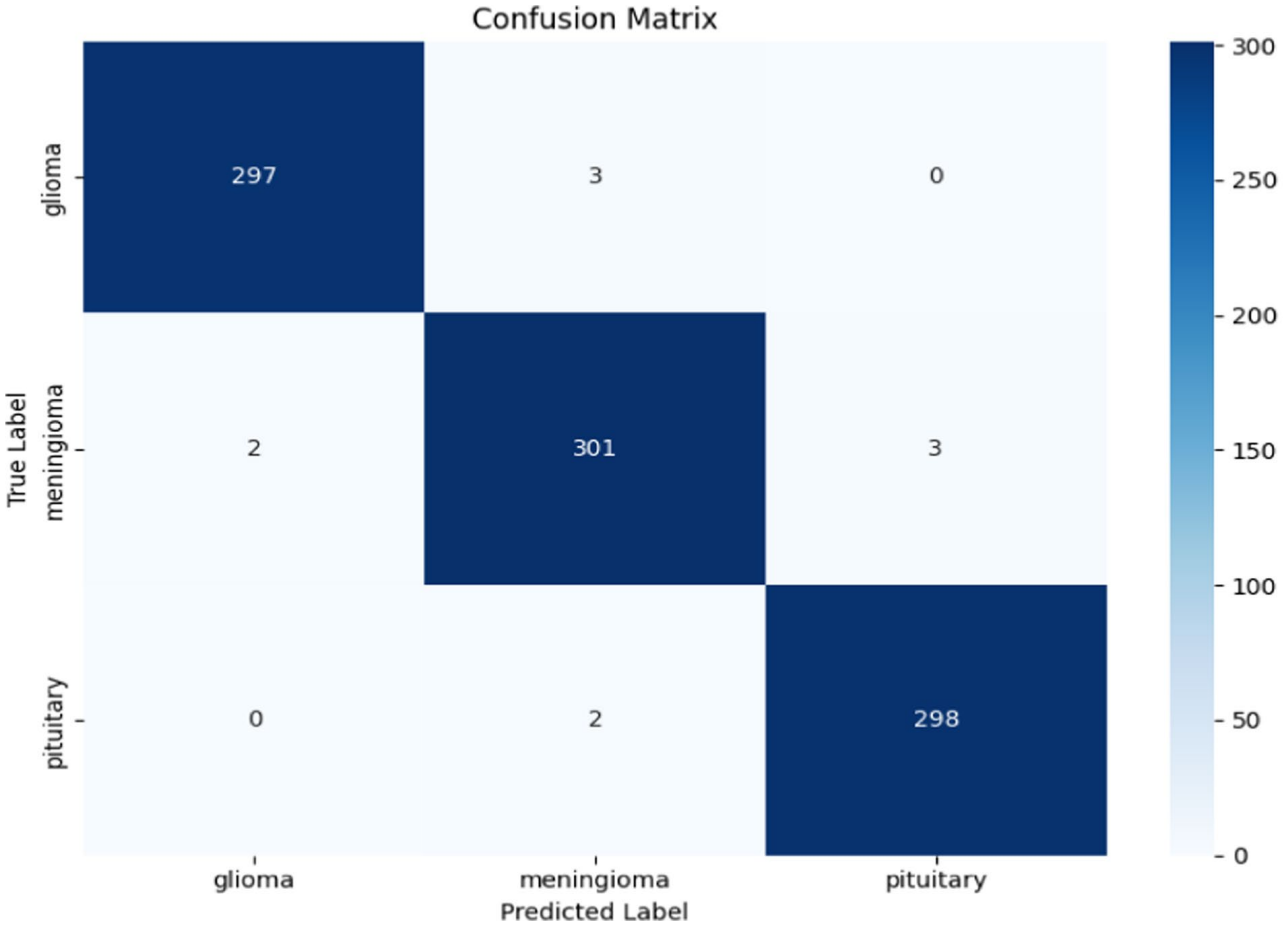
Confusion matrix for the hybrid cGAN and AE + Swin Transformer model.

The confusion matrix in Figure 24 provides a complete summary of the classification results on the test set and shows that the overall accuracy is high across all classes. The model performed best in the ``no tumour” class, where all samples were correctly classified. In the instances where the model appeared to misclassify cases, it was mostly between glioma and meningioma, which is expected given how similar they are when viewed using MRI scan or photographs. Overall, while there is likely some overlap between categories in terms of patient health, the model is successful at categorizing a case into the major tumour categories versus healthy diagnosis with considerable precision.

**Figure 25:**
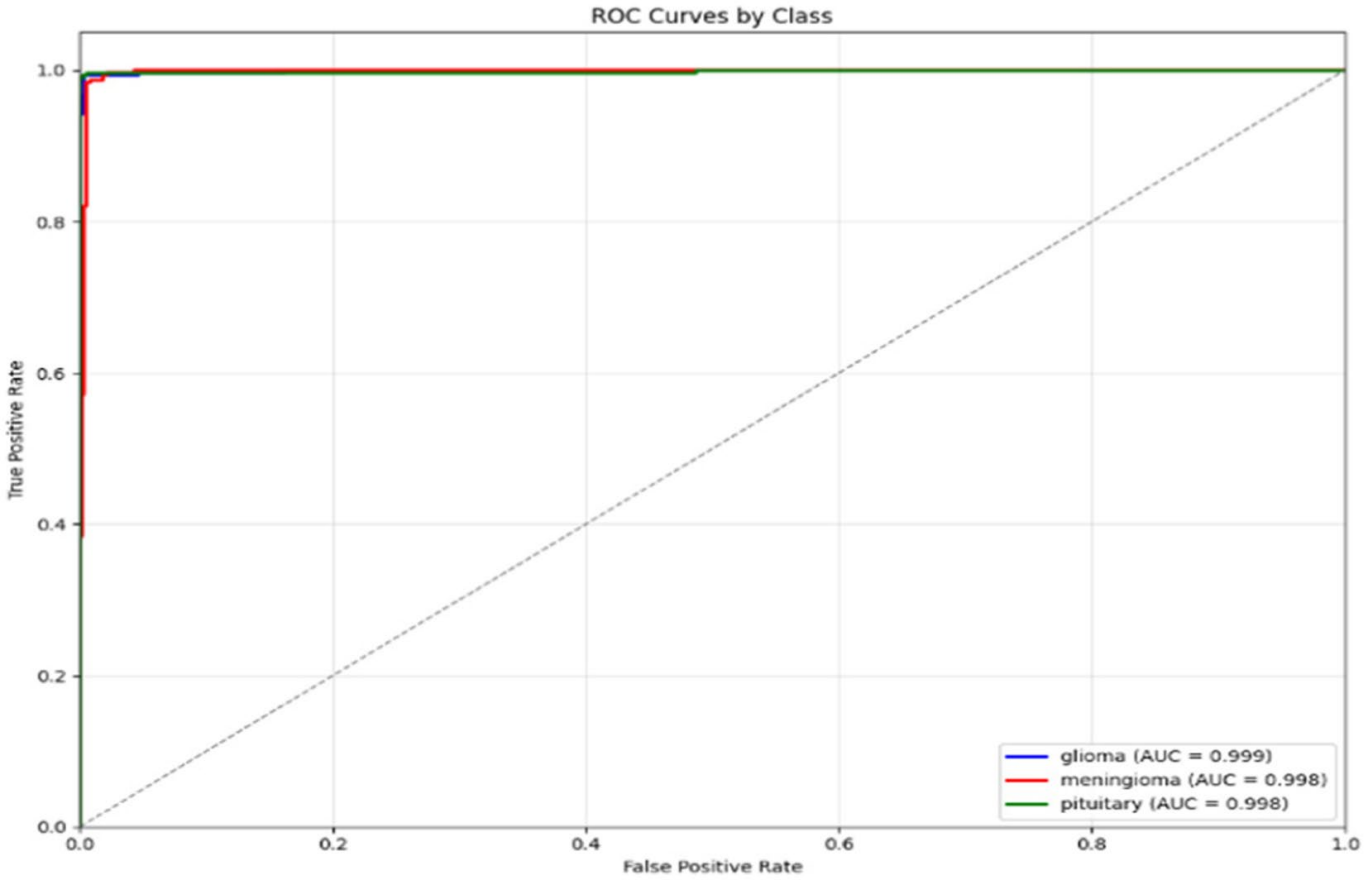
ROC curves for glioma, meningioma, and pituitary classes.

In Figure 25, the Receiver Operating Characteristic (ROC) curves for the classification model for three tumour types are shown: glioma, meningioma, and pituitary. The ROC curves plot the True Positive Rate (TPR) versus the False Positive Rate (FPR), and each curve correlates to one tumour class. The area under the curve (AUC) is reported for each tumour class, which indicates how well the model can differentiate between the tumour classes. In this case, the model has high AUC for all three tumour types (all close to 1.0), where glioma (AUC = 0.999) is the best model, then meningioma and pituitary (AUC = 0.998). Overall, these results demonstrate that the model is excellent at distinguishing the tumour differences.

**Figure 26:**
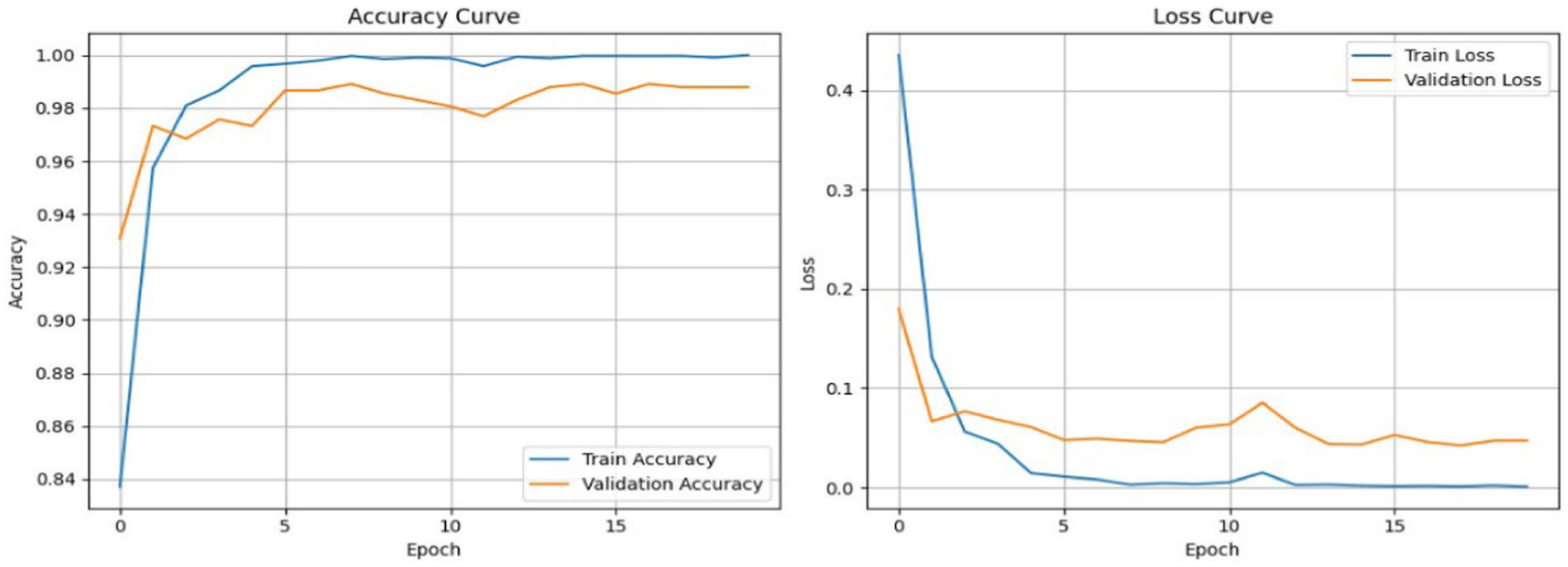
Training vs. validation accuracy and loss curves for Dataset II.

To investigate training stability, Figure 26 shows the training and validation accuracy and loss curves for 20 epochs. The plots indicate solid and stable convergence with minimal generalization gap between training and validation. Both of the loss curves steadily decrease, while the accuracy curves steadily increase before reaching a plateau which emphasizes rapid learning and overall model stability. This observation demonstrates the effectiveness of the synthetic data augmentation approach and the Swin Transformer’s ability to generalize very well, even with a multiclass imbalanced medical dataset.

### 3.4 Cross validation

This section presents the results of the cross-validation process using Dataset I and Dataset II. The model was evaluated on validation and test sets for all three folds. The results for each fold, along with the mean and standard deviation of the accuracies are given in the Table 5 below.

**Table 5:** Validattion Performance of Dataset I.

Fold	Validation accuracy	Test accuracy
1	98.53	99.08
2	99.05	99.24
3	99.21	99.31
Mean	98.93	99.21
Std dev	0.29	0.10

The model consistently obtained high performance through all three folds, with validation accuracies between 98.53 and 99.21%, while the test accuracies ranged from 99.08 to 99.31%, illustrating the model’s ability to generalize unseen data. The Table 6 presents the performance of the model evaluated through cross-validation using Dataset II.

**Table 6:** Validattion performance Dataset II.

Fold	Validation accuracy	Test accuracy
1	99.05	99.22
2	98.68	98.56
3	98.83	98.23
Mean	98.88	98.67
Std Dev	0.0015	0.004

The above table presents the performance of the model through 3-fold cross-validation, showing both validation and test accuracies for each fold. The model achieves high and consistent performance, with validation accuracies ranging from 98.68 to 99.05%, and test accuracies from 98.23 to 99.22%. The mean validation accuracy is 98.88%, while the mean test accuracy is 98.67%, with low standard deviations (0.0015 for validation and 0.004 for test), indicating stable and reliable performance across all folds.

### 3.5 Comparison with existing work

To evaluate the effectiveness of the proposed approach, we compare its performance with prior deep learning-based brain tumour classification models reported in recent literature. The comparisons are made separately for Dataset I and Dataset II, and are based on key metrics such as accuracy, precision, recall, F1-score, and model complexity when available. Table 7 summarizes the accuracy achieved by various models on Dataset I. The proposed method surpasses most existing works in terms of overall accuracy, while also demonstrating excellent class-wise performance and generalization.

**Table 7:** Comparison of performance on Dataset I.

References	Method	Accuracy (%)
(47)	Multi-Level GAN + CNN	96.00
(48)	EfficientNetB3 + SE + Inverted Bottlenecks	97.50
(49)	PDCNN with Preprocessing (Binary)	98.12
(59)	Dilated PDCNN + Ensemble + ML Classifiers	98.35
(50)	Optimized Ensemble Network	98.00
(51)	Bayesian Depth-Wise CNN	94.38
(52)	DenseNet, Modified CNN	90.30, 99.14
(49)	PDCNN	97.33
(53)	Transfer Learning utilizing pre-trained neural networks: ResNet-50	99
Proposed (This Work)	Swin Transformer + AE-GAN Augmentation	99.54

The table compares the proposed model, which is a combination of Swin Transformer and AE-GAN Augmentation, with a number of state-of-the-art methods. The proposed model achieves 99.54\% accuracy, which is better than any of the other methods mentioned above. Asiri et al. (47) with the Multi-Level GAN + CNN combination achieved 96.00% accuracy scores, while Reyes and Sánchez (48) with EfficientNetB3 reached 97.50%. The fourth-place accuracy came from Rahman et al. (49) with a Dilated PDCNN + Ensemble method, where they had an accuracy of 98.35%. Other methods, such as the one by Hekmat et al. (50), had accuracies between 94.38–98.00%, including Ekong et al. (51). In the research conducted by Osman Özkaraca et al. (52), the DenseNet model achieved a test accuracy of 99.14%, while the Modified CNN model had a test accuracy of 94.55% in the brain tumor classification task. In the study by Rahman and Islam (49) the PDCNN (Parallel Deep Convolutional Neural Network) model achieved a test accuracy of 97.33% for brain tumor classification. In the study by Nurtay et al. (53) Transfer Learning utilizing pre-trained neural networks, specifically ResNet-50, achieved a test accuracy of 99% in brain tumor classification.

The significantly better performance of the proposed model highlights the power of incorporating a Swin Transformer and generating synthetic datasets as augmentation to enhance model performance, especially when working with complex and imbalanced datasets.

Table 8 shows the accuracy of a number of models on a likely medical image classification task. The proposed GAS Model, combining the Swin Transformer and AE-GAN augmentation, achieved 98.90\% accuracy the highest out of all the models reviewed.

**Table 8:** Comparison of classification accuracy on Dataset II.

References	Method	Accuracy (%)
(54)	GoogLeNet + KNN	98.30
(55)	Two-Channel Deep CNN	95.23
(56)	Hybrid CNN Ensemble	96.08
(57)	Deep CNN	97.27
(58)	ResNet-18 Feature Extraction + SVM	98.62
(49)	ResNet50 (Transfer Learning), EfficientNetB0	98.4, 94.1
(57)	Deep CNN	97.27
Proposed Model	Swin Transformer + AE-GAN Augmentation	98.90

In comparison, Sekhar et al. (54) with GoogLeNet + KNN achieved 98.30% accuracy, and Bodapati et al. (55) with a Two-Channel Deep CNN achieved 95.23%. Singh et al. (56) with a Hybrid CNN Ensemble reached 96.08%, and Sadr et al. (57) using a Deep CNN achieved 97.27%. The model proposed by Alrikabi et al. (58) using ResNet-18 as a feature extractor followed by an SVM classifier achieved 98.62% accuracy. Sadr et al. (57) found that the ResNet50 (Transfer Learning) model had an accuracy of 98.4%, and the EfficientNetB0 model had an accuracy of 94.1%. However, Sadr et al. (57) used a Deep CNN model that had an accuracy of 97.27%.

The comparison results show that the proposed model with the Swin Transformer and AE-GAN augmentation yielded superior accuracy compared to the other methods and seems to be a very effective approach to achieve high accuracy and generalize results, especially with the inclusion of synthetic data augmentation.

## 4 Ablation study

### 4.1 Swin Transformer by sing Dataset I

This experiment assesses the performance of the Swin Transformer model on Dataset I, which contains MRI images labelled with four classes of tumor: Notumor, Meningioma, Glioma, and Pituitary. The primary objective of this experiment is to evaluate how well the Swin Transformer, a state-of-the-art vision transformer model for performing classification from images, recognizes different types of brain tumors through MRI images. This experiment was conceived in a way that it only tests the ability of the Swin Transformer model to recognize and distinguish between these tumor classes limited only to raw MRI images, and exclude possible confounds of any data augmentation or pre-processing methods, such as AE-cGAN (see Table 9).

**Table 9:** Classification performance of the Swin Transformer Model on Dataset I.

Class	Precision (%)	Recall (%)	F1-Score (%)	Support	Accuracy	Macro avg	Weighted avg
Notumor	100	100	100	405			
Meningioma	100	99	99	300			
Glioma	98	99	98	306			
Pituitary	99	100	99	300			
Overall				1,311	99%	99%	99%

The Swin Transformer Model’s performance was exceptional in classifying brain tumors, achieving a 99% overall accuracy rate. The model was able to correctly identify all Notumor cases (100% precision and recall) and had precision and recall for Meningioma and Pituitary tumors close to 100% indicating practically flawless classifications. For Glioma, the Swin had a lower precision (98%), however still performed well with high recall (99%) and an F1-score of 98%. The precision, recall, and F1-score for both the macro average and weighted average metric were all 99% percent confirming that the Swin consistently performed excellent and in a reliable manner across all tumor types demonstrating its clearly considerable power in group and twin-class medical photo classification tasks.

### 4.2 Swin Transformer and cGAN by sing Dataset I

In this ablation study, we evaluate the performance of the Swin Transformer combined with cGAN using Dataset I. This setup aims to explore the contribution of both the Swin Transformer for classification and the cGAN for generating synthetic data to enhance the model’s ability to handle data imbalance. By integrating cGAN-generated synthetic images, we aim to observe whether this augmentation improves the model’s generalization and classification performance compared to using the Swin Transformer alone. The results of this study will help determine the individual and combined effectiveness of the Swin Transformer and cGAN in improving classification accuracy for brain tumor detection.

The Table 10 shows the performance of the model in classifying tumors on Dataset I. Overall, the model had an accuracy of 99.1% on the various brain tumor types and performed very well on the diversity of tumors. The notumor classification was perfect with precision, recall, and F1 all equal to 1.0. Meningioma had a minor reduction in recall to 0.99 resulting in a F1 score of 99%. Glioma had precision, recall, and F1 scores around 0.99. Pituitary tumors had 98% precision and perfect recall resulting in an F1 score of 99%. The macro average and weighted average were the same at 99.1%. Overall, these scores indicate consistent performance of the model across all classes.

**Table 10:** Classification report for Swin Transformer and cGAN Model on Dataset I.

Class	Precision (%)	Recall (%)	F1-Score (%)	Support	Accuracy	Macro avg	weighted avg
Notumor	100	100	100	405			
Meningioma	100	99	99	300			
Glioma	99.1	99.1	99	306			
Pituitary	98	100	99	300			
Overall				1,311	99.1%	99.1%	99.1%

### 4.3 Swin Transformer by sing Dataset II

In this ablation study, we evaluate the performance of the Swin Transformer using Dataset II.

The Table 11 shows the classification performance of the model for three types of brain tumors (Glioma, Meningioma and Pituitary). The overall accuracy was 95.8%, which indicates that the model is strong in classification ability. The model achieved a precision of 97.8% and recall of 93.0% for Glioma, resulting in an F1-score of 95.3%. For Meningioma the precision was 97.2% and recall was 97.2%, therefore an F1-score of 97.2%. The model also performed well on Pituitary, achieving a precision of 92.0% and a recall of 98.9%, yielding an F1-score of 95.3%. The macro average and weighted average for precision, recall and F1-score were 95.7 and 95.9%, respectively, which shows that the classification performance for each class is reliable and consistent. Thus, it can be concluded that the model can classify brain tumors with very few misclassifications.

**Table 11:** Classification performance of the Swin Transformer model on Dataset II.

Class	Precision (%)	Recall (%)	F1-score (%)	Support	Accuracy (%)	Macro avg (%)	Weighted avg (%)
Glioma	97.8	93.0	95.3	143			
Meningioma	97.2	97.2	97.2	71			
Pituitary	92.0	98.9	95.3	93			
Overall				307	95.8	95.7	95.9

### 4.4 Swin Transformer and cGAN by sing Dataset II

In this ablation study, we evaluate the performance of the Swin Transformer combined with cGAN using Dataset II.

The table details the classification performance of the Swin + GAN Model on Dataset I with a model overall accuracy of 99%. For Class 0 (Notumor), the model exhibited a precision of 99% and a recall of 98%, resulting in an F1-score of 98%. This indicates the model had strong performance identifying non-tumor images, but was likely to miss a small number of true non-tumor images. For Class 1 (Meningioma), the model achieved a precision of 97%, and a recall of 99% resulting in a F1-score of 98%. The model started off with a slightly higher number of false positives, but still provided excellent identification of true meningioma cases. For Class 2 (Pituitary), the model had a precision of 100% and a recall of 99%, which resulted in an F1-score of 99% that excelled classification of pituitary tumor images. Overall accuracy and the macro and weighted averages for precision, recall, and F1-score was 99%, providing elegant consistent performance across all tumor types, without significant misclassification of each tumor type (see Table 12).

**Table 12:** Classification report for Swin Transformer and cGAN model on Dataset II.

Class	Precision (%)	Recall (%)	F1-Score (%)	Support	Accuracy (%)	Macro avg (%)	Weighted avg (%)
0	99	98	98	300			
1	97	99	98	306			
2	100	99	99	300			
Overall				906	99	99	99

## 5 Discussion

The proposed model with AE in conjunction with Swin Transformer and cGAN addresses many complexities of brain tumour classification, specifically the issues surrounding data scarcity. The inclusion of cGAN substantially improves the proposed model in generating synthetic data, which expands the dataset the divider can train on in situations where there is insufficient labelled data. Generating data using cGAN can drastically enhance the model’s generalization ability, especially in cases of an imbalanced dataset. The model will be able to generate synthetic MRI images of high-quality, with complexities and variations that can represent real medical images, allowing the competition to train on a more complex and balanced dataset, improving classification accuracy and robustness.

The incorporation of cGAN leads to improved performance in contrast to standard GANs, because it is a conditional network that generates images closely related to the target tumour types. Consequently, cGAN creates more realistic and representative synthetic samples that improve model classification performance on complex tumour types (e.g., glioma, meningioma, and pituitary tumours) by creating high precision and recall. Additionally, the Swin Transformer can improve performance for classification of high-resolution medical images, because the hierarchical attention mechanism allows for effective modeling of both local and global features.

On Dataset I, our model achieves an impressive accuracy of 99.54% compared to Dataset II with an accuracy of 98.9%. This performance also beats several state-of-the-art approaches to classification and has potential clinical impact. The hybrid architecture included AE for feature extraction, cGAN for data augmentation, and Swin Transformer for classification, and ensures that component’s strengths and weaknesses are acknowledged to focus on critical issues for medical imaging analysis. The results show that cGAN has an important role to play in improving classification performance by providing large amounts of high-quality synthetic data. This is especially important in real-world clinical applications where obtaining large annotated datasets can be the bottleneck.

The proposed model, while demonstrating accuracy for classifying brain tumors, will face challenges in its transition into health care. The model must be fit into existing clinical work flows, such as, MRI processing systems, and electronic health records, and be reliable, accurate, and explainable within resource-constrained environments. It would also require regulatory approval, for example, FDA clearance, as well as conformance with data privacy regulations such as HIPAA and GDPR. Furthermore, explainable AI is essential for use in clinical practice. Clinicians will need to be provided with explanations justifying the model’s predictions in order to make suitable clinical judgments. This could take the form of opacity tools such as saliency maps or attention visualization to aid interpretation. These barriers will likely need to be addressed in collaboration with clinicians and the model will need to be proved reliable and explainable. Ultimately, when this is achieved in practice it is hoped that it will augment, rather than replace, clinical judgment in the context of real-world epidemics.

In the future, we plan on augmenting the dataset, especially with additional representatives and diversities in subject details and more representative or balancing of examples, and have the capability to create a custom portal tools or web-based system (i.e., application that allows real time and automatic learning processes). This will further enhance post-hoc adjustments for analyzing and improving the model performance before the model can attain learning autonomy.

## 6 Conclusion

The study proposed a new hybrid model with AE, cGAN, and Swin Transformers to handle the challenges in brain tumour classification; such as data imbalance and lack of annotated datasets. By implementing cGAN’s ability to generate synthetic data, we were able to augment some cases that the model did poorly in with synthetic data to improve generalization and accuracy in the classification. Results achieved in this study were extraordinary with classification accuracy 99.54% and 98.9% on two publicly available datasets.

These results demonstrate the efficacy of combining a feature extraction model, data augmentation methodology using synthetic data generation, and an advanced architecture using Swin Transformers for image classification, while supporting the claim that this model can outperform traditional manual approaches. Our future research aims to adapt the proposed model for clinical usage with capabilities for real-time assessments. Further development will involve integrating multimodal imaging sources within the dataset.

Future endeavors will be directed towards clinical deployment in real-time and optimizing the model for faster inference times, while also ensuring seamless integration within existing healthcare infrastructure. Furthermore, the model can be extended to incorporate multi-modal imaging data, including T1, T2, FLAIR MRI scans, or even CT scans to further enhance its robustness. Development will also center around enhancing the quality of synthetic data produced by the cGAN in a way that fails and will rely on handling rare and complex tumors. Exploring transfer learning and fine-tuning on other datasets can allow for improved generalizations to new, unseen data.

Also, to further improve accuracy, we will focus on obtaining and developing better quality synthetic data, and testing the models in a clinical setting to study model’s interpretability, providing the model with boundaries of use to enhance robustness, transparency, and applicability. Ultimately, the goal was to improve speed and accuracy in diagnosing brain tumours, with restrictions to address for clinical diagnosis to enhance the precision of the detection process and ultimately more accurate and timely diagnoses.

## Data Availability

The original contributions presented in the study are included in the article/supplementary material, further inquiries can be directed to the corresponding author.

## Glossary

G: Generator in Conditional GAN
D: Discriminator in Conditional GAN
AE: Autoencoder
E (x): Encoder function of the Autoencoder, mapping input image xx to latent space
f_encoder_: Encoder function used in both Autoencoder and cGAN Discriminator
x: Input image
y: Class label
z: Latent feature space representation
G(z, y): Synthetic image generated by the Generator conditioned on zz and yy
L_D_: Discriminator loss (binary cross-entropy for real vs. fake classification)
L_G_: Generator loss (encouraging the generator to produce realistic images)
Pre: Precision (the proportion of true positives over all predicted positives)
Rec: Recall (the proportion of true positives over all actual positives)
F_1_-S: F1-Score (harmonic mean of Precision and Recall)
L_CE_: Cross-Entropy Loss for multi-class classification
AUC: Area Under the Curve (used in ROC curve analysis)
Swin-Tiny: Pretrained Swin Transformer model used for classification
ŷ: Predicted label (output of the classifier)
